# Glioma glycolipid metabolism: MSI2–SNORD12B–FIP1L1–ZBTB4 feedback loop as a potential treatment target

**DOI:** 10.1002/ctm2.411

**Published:** 2021-05-12

**Authors:** Weiwei Dong, Xiaobai Liu, Chunqing Yang, Di Wang, Yixue Xue, Xuelei Ruan, Mengyang Zhang, Jian Song, Heng Cai, Jian Zheng, Yunhui Liu

**Affiliations:** ^1^ Department of Neurosurgery Shengjing Hospital of China Medical University Shenyang China; ^2^ Key Laboratory of Neuro‐oncology in Liaoning Province Shenyang China; ^3^ Liaoning Province Medical Surgery and Rehabilitation Robot Technology Engineering Research Center Shenyang China; ^4^ Department of Neurobiology, School of Life Sciences China Medical University Shenyang China; ^5^ Key Laboratory of Cell Biology, Ministry of Public Health of China China Medical University Shenyang China; ^6^ Key Laboratory of Medical Cell Biology, Ministry of Education of China China Medical University Shenyang China

**Keywords:** alternative polyadenylation, glioma, glycolipid metabolism, molecular pattern, MSI2, potential target, prediction and personalized prevention, SNORD12B, translational research, ZBTB4

## Abstract

Abnormal energy metabolism, including enhanced aerobic glycolysis and lipid synthesis, is a well‐established feature of glioblastoma (GBM) cells. Thus, targeting the cellular glycolipid metabolism can be a feasible therapeutic strategy for GBM. This study aimed to evaluate the roles of MSI2, SNORD12B, and ZBTB4 in regulating the glycolipid metabolism and proliferation of GBM cells. MSI2 and SNORD12B expression was significantly upregulated and ZBTB4 expression was significantly low in GBM tissues and cells. Knockdown of MSI2 or SNORD12B or overexpression of ZBTB4 inhibited GBM cell glycolipid metabolism and proliferation. MSI2 may improve SNORD12B expression by increasing its stability. Importantly, SNORD12B increased utilization of the ZBTB4 mRNA transcript distal polyadenylation signal in alternative polyadenylation processing by competitively combining with FIP1L1, which decreased ZBTB4 expression because of the increased proportion of the 3′ untranslated region long transcript. ZBTB4 transcriptionally suppressed the expression of HK2 and ACLY by binding directly to the promoter regions. Additionally, ZBTB4 bound the MSI promoter region to transcriptionally suppress MSI2 expression, thereby forming an MSI2/SNORD12B/FIP1L1/ZBTB4 feedback loop to regulate the glycolipid metabolism and proliferation of GBM cells. In conclusion, MSI2 increased the stability of SNORD12B, which regulated ZBTB4 alternative polyadenylation processing by competitively binding to FIP1L1. Thus, the MSI2/SNORD12B/FIP1L1/ZBTB4 positive feedback loop plays a crucial role in regulating the glycolipid metabolism of GBM cells and provides a potential drug target for glioma treatment.

## Graphical abstract

1. MSI2 expression was significantly upregulated in GBM, binding to SNORD12B and improving SNORD12B expression by increasing its stability.

2. SNORD12B regulated alternative polyadenylation process of transcriptional repressor ZBTB4 by competitively combining with FIP1L1.

3. ZBTB4 transcriptionally suppressed the expression of HK2, ACLY, and MSI2, forming a positive feedback regulatory loop to regulate the glycolipid metabolism and proliferation of GBM cell.

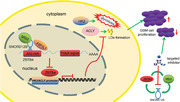

## 1 INTRODUCTION

### 1.1 Glioblastoma as a major medical challenge

Glioblastoma (GBM) is the most prevalent and malignant primary intracranial tumor.^1^ Although multiple treatments and their combinations are available, such as surgery, radiation therapy, and molecular target therapy, the prognosis of patients with GBM remains unfavorable. The median survival time of GBM patients is approximately 12–15 months.^2^ Reprogramming of energy metabolism is an important indicator of malignant tumors.^3^, ^4^, ^5^ Abnormal energy metabolism has also been reported in GBM and includes enhanced aerobic glycolysis and lipid synthesis to provide energy and raw materials to the highly proliferating cells.^6^, ^7^, ^8^, ^9^ Thus, key glycolipid metabolic enzymes and transcription factors are considered promising treatment targets and diagnostic and prognostic tumor markers for predictive, preventive, and personalized medicine.^10^ Therefore, we proposed that targeting the cellular energy metabolism may be a new strategy for treating GBM.

### 1.2 RNA‐binding proteins and their role in glioma tumorigenesis

RNA‐binding proteins (RBPs) are widely involved in the posttranscriptional modification of RNA, including RNA splicing, RNA editing, and alternative polyadenylation (APA), and extensively participate in tumorigenesis.^11^ For example, RBP‐ANKHD1 expression is upregulated and promotes angiogenesis in GBM.^12^ RBP‐Lin28A enhances the proliferation of GBM cells by improving the aerobic glycolytic capacity.^9^ A recent study showed that Musashi RNA‐binding protein 2 (MSI2) is highly expressed in GBM and stimulates the ability of GBM cells in proliferation, migration, and invasion. It also participates in regulating the resistance of GBM cells to temozolomide chemotherapy.^13^ However, the effect of MSI2 on energy metabolism in GBM cells remains unknown.

### 1.3 SnoRNAs as novel molecular targets for cancer treatment

Previous studies have mainly investigated the value of miRNAs in tumor‐targeted treatments, diagnostics, and prognostics.^14^ Recent studies have found that small nucleolar RNAs (snoRNAs), a type of small noncoding RNAs that are localized in the nucleus, play a regulatory role in tumorigenesis. For instance, SNORA72 displays high expression in ovarian cancer and promotes the maintenance of ovarian cancer stem cell stemness by activating the Notch1/c‐Myc pathway.^15^ In colon cancer, SNORD16 promotes tumorigenesis, and SNORD16 knockdown restrains the proliferated, migrated, and invaded ability of colon cancer cells and promotes apoptosis.^16^ SNORD12B is formed from the splicing intron of the long noncoding RNA ZFAS1. SNORD12B expression is upregulated in colon cancer and is correlated with its metastasis.^17^

### 1.4 Activity of zinc finger and BTB domain‐containing 4 in tumors

Zinc finger and BTB domain‐containing 4 (ZBTB4) is a transcription factor and a member of the zinc finger protein family. Recent studies found that ZBTB4 mainly acts as a transcription inhibitor in tumor cells. ZBTB4 functions as an antioncogene in Ewing's sarcoma by regulating the cell cycle and promoting apoptosis.^18^ In breast cancer, ZBTB4 can inhibit oncogene transcriptional activity, and its overexpression suppresses the growth and migration of cancer cells.^19^, ^20^ However, the regulatory effects of SNORD12B and ZBTB4 in the glycolipid metabolism and proliferation of GBM cells remain unclear.

### 1.5 Key enzymes in glioma glycolipid metabolism

Hexokinase‐2 (HK2), the first enzyme committed to glycolysis, converts glucose to glucose‐6‐phosphate, a key mediator of aerobic glycolysis. Compared with normal cells, tumor cells show dramatically augmented glucose utilization and lactate production. HK2 is significantly overexpressed in various tumors, and its knockdown can suppress the aerobic glycolytic rate and proliferation of cancer cells.^21^ Thus, an increasing number of studies on cancer metabolism are examining the regulation of HK2 expression as a promising anticancer strategy.^10^, ^21^, ^22^ HK2 contributes to increasing the glycolytic capacity and proliferation of GBM cells.^23^ Human ATP citrate lyase (ACLY) catalyzes the ATP‐dependent synthesis of oxaloacetate and acetyl‐coenzyme A (acetyl‐CoA) from citrate and coenzyme A (CoA), which is a key enzyme in lipid synthesis. ACLY is generally overexpressed in tumor cells, and its knockdown inhibits lipid synthesis in lung and prostate cancer cells, as well as tumor growth.^24^, ^25^, ^26^ A recent study demonstrated that ACLY expression is significantly upregulated in GBM cells and that it participates in the regulation of energy metabolism and cell proliferation.^27^, ^28^

In this study, we explored the endogenous expression of MSI2, SNORD12B, and ZBTB4 in GBM tissues and cells. Further, we evaluated the interactive model of these molecules and clarified their roles in glycolipid metabolism and the proliferation of GBM cells. This study aimed to reveal a new molecular pathway regulating cancer energy metabolism and provide new potential molecular targets for GBM therapy.

## 2 MATERIALS AND METHODS

### 2.1 Clinical specimens

The normal brain and glioma samples were obtained from the Department of Neurosurgery in Shengjing Hospital Affiliated China Medical University. All patients signed informed consent, which received ethical approval from the Ethics Committee of Shengjing Hospital Affiliated China Medical University. For more details, please see Supporting Information of Materials and Methods.

### 2.2 Cell culture

Human embryonic kidney (HEK) 293 T cells and glioma cell lines (U251 and U373) were ordered from Institutes for Biological Sciences Cell Resource Center (Shanghai, China). Normal human astrocyte (NHA) was ordered from the ScienCell Research Laboratories (CA, USA). For more details, please see Supporting Information of Materials and Methods.

### 2.3 RNA extraction and quantitative real‐time PCR

RNA expression levels in present study were measured by the means of quantitative real‐time PCR (qRT‐PCR). qRT‐PCR was operated as previously described.^29^ Total RNA was extracted from cells and tissues by utilizing Trizol reagent (Life Technologies Corporation, Carlsbad, CA, USA). The primers are displayed in Table S1. For more details, please see Supporting Information of Materials and Methods.

### 2.4 Cell transfection

The short‐hairpin RNA against MSI2(MSI2(−)) and its nontargeting sequence (MSI2(−)NC); the short‐hairpin RNA against ZBTB4 (ZBTB4(−)), ZBTB4 full length (ZBTB4(+)) sequence and their respective negative control (ZBTB4(−)NC or ZBTB4(+)NC) plasmids; SNORD12B full length (SNORD12B(+)) sequence and its negative control (SNORD12B(+)NC) plasmids were synthesized by Gene‐Pharama (Shanghai, China). The silencing plasmids targeting sequences of MSI2 and ZBTB4 are shown in Table S2. SNORD12B sgRNA CRISPR/Cas9 All‐in‐One plasmid vector set was used to inhibit SNORD12B expression. CRISPR/Cas9 target sequences of SNORD12B (SNORD12B(−)) and recommended scrambled control (SNORD12B(−)NC) were synthesized by Syngen Tech (Beijing, China). The sgRNA for knockdown of SNORD12B is shown in Table S3. The qRT‐PCR or Western blot was performed to detect transfection efficacy (Figure S2). For more details, please see Supporting Information of Materials and Methods.

### 2.5 Western blot

Western blot was operated as previously described.^29^ For details of the experiment and antibodies used, please see Supporting Information of Materials and Methods.

### 2.6 Measurement of extracellular acidification rate

Extracellular acidification rate (ECAR) was performed as previously described.^9^ For more details, please see Supporting Information of Materials and Methods.

### 2.7 Metabolic parameters of glucose utilization and production of lactate

The assay was performed using lactate assay kit (Jiancheng, Nanjing, China) and glucose assay kit (Rsbio, Shanghai, China). Briefly, 2 × 10^4^ targeted cells per well were seeded in 96‐well plate with 200 μl medium. After 48 h culturing, the supernatant culture medium was collected and lactate or glucose level was detected using colorimetric method following the manufacturer's protocol. The cell number was used to normalize lactate production and glucose consumption rate.

### 2.8 Dual‐luciferase reporter assay

The assay was operated as previously described.^29^ For more details, please see Supporting Information of Materials and Methods.

### 2.9 RNA electrophoretic mobility shift assay

The cell nuclear was isolated by using nuclear extraction kit (Thermo Fisher, Carlsbad, CA, USA). The EMSA assay was carried out using LightShift EMSA kit (Thermo Fisher, Carlsbad, CA, USA) according to the manufacturer's instructions. The nuclear protein extraction, biotin‐labeled oligonucleotide probe and 1 μl ZBTB4 antibody (Proteintech, Chicago, IL, USA) were added into reaction system. Unlabeled oligonucleotide acted as competitor, which can eliminate the shift band by competitive binding of the labeled probe or target protein. After 20‐min incubation, the mixture was subjected to 5% polyacrylamide gel electrophoresis and the band was transferred to a Nylon membrane (Thermo Fisher, Carlsbad, CA, USA). Then, the membrane was exposed to UV irradiation to crosslink RNA‐protein complexes into membrane. The membrane was incubated with HRP‐conjugated streptavidin and visualized with Chemiluminescent EMSA Kit (Beyotime Institute of Biotechnology, Jiangsu, China) and scanned by ChemImager 5500 V2.03 software.

### 2.10 Analysis of distal poly(A) site usage

To analyze the distal poly(A) site usage (dPAS), we designed the common primer to target ZBTB4 open reading frame and normalized for total transcript. The distal primers were designed to the target sequence just before the dPAS to explore the long transcript of ZBTB4, which was generated by using the dPAS. All primers are shown in Table S1. The percentage of dPAS utilization was calculated as ΔCT = CT_distal_ − CT_total_. Data were shown as fold changes normalized to control by calculating ΔΔCT = ΔCT_average target_ − ΔCT_average control_.

### 2.11 Rapid amplification of cDNA end and Sanger sequencing

Rapid amplification of cDNA end (3′‐RACE) was conducted using 3′‐Full RACE Core Set with PrimeScript RTase (Takara Bio Inc., Kusatsu, Japan) to identify the 3′UTR of ZBTB4. The Gene‐Specific Primer 1 (GSP1, first round, TCTACTCTTCTGGTGGGCTTTG), GSP2 (second round, CCCTGCTCTGTGATTGGATAA) were designed to target the last exon of ZBTB4. The RNA was reversed‐transcribed into cDNA by using PrimeScript RTase with 3′‐RACE adaptor (including dT region and adaptor primer sequence). The Outer 3′‐RACE PCR was performed by using LA‐Taq (Takara Bio Inc., Kusatsu, Japan) with GSP1 and 3′‐RACE outer primer. The 3′‐RACE PCR products were diluted at 1:25 in nuclease‐free water and used as templates for inter‐nested 3′RACE PCR. Inter‐nested 3′RACE PCR was performed using GSP2 and 3′‐RACE inner primer. The final PCR products were purified and 3′UTR variants were identified by Sanger sequencing.

### 2.12 RNA immunoprecipitation assay

The RNA immunoprecipitation (RIP) assay was operated as previously described.^9^ For more details, please see Supporting Information of Materials and Methods.

### 2.13 RNA pull‐down assay

The assay was operated as previously described.^9^ For more details, please see Supporting Information of Materials and Methods.

### 2.14 Lipid droplet staining and quantification

The lipid droplets were stained using BODIPY 493/503 (Glpbio, Montclair, CA, USA). The cells were plated on sterile glass coverslips and cultured until 50% confluence before staining. The cells were rinsed twice with PBS and fixed with paraformaldehyde for 30 min at room temperature. Lipid droplets were stained with BODIPY493/503 (0.5 μM) for 30 min and washed with PBS three times. Then, the nucleus was counterstained with 0.5 mg/ml DAPI (Beyotime, Jiangsu, China) for 5 min and washed with PBS three times. The staining was visualized by confocal microscopy. More than 15 cells were analyzed and lipid droplet numbers were quantified with the Image‐J software.

### 2.15 Colorimetric triglyceride and cholesterol measurement assay

Triglyceride was measured using commercial triglyceride quantification assay kit (Jiancheng, Nanjing, China). In brief, triglyceride was converted to glycerol and free fatty acid when lipase was added. Then, the glycerol was oxidized following glycerol kinase and oxidase added successively, and finally yielded H_2_O_2_, which reacted with commercial probe to produce color (OD 510 nm). Similarly, total cholesterol was measured using commercial cholesterol quantification assay kit (Jiancheng, Nanjing, China). Cholesterol was oxidized by enzyme provided by assay kit and finally generated H_2_O_2_, then, H_2_O_2_ reacted with commercial probe to produce color (OD 510 nm). Absorbance was detected at a wavelength of 510 nm via SpectraMax M5 microplate reader (Molecular Devices, USA). The final level of triglyceride or cholesterol was normalized by protein concentration, which was detected by BCA protein assay.

### 2.16 Cell viability assay

The assay was operated as previously described.^29^ For more details, please see Supporting Information of Materials and Methods.

### 2.17 Gene expression profile microarray

The stable MSI2 knockdown and corresponding NC glioma cells were used for the expression profile of snoRNA. Briefly, total RNA was extracted from each sample on the basis of the manufacturer's protocol mentioned above, and the RNA extracts were evaluated by Nanodrop Spectrophotometer (ND‐100, Thermo, USA), and the ratio of A260/A280 was close to 2.0. Then RNA extracts were amplified and transcribed into cDNA using rtStar First‐Strand cDNA Synthesis Kit (KangChen Bio‐Tech Inc., Shanghai, China). Lastly, the microarray assay was performed using nrStar snoRNA PCR microarray (KangChen Bio‐Tech Inc., Shanghai, China).

The stable SNORD12B knockdown and corresponding NC glioma cells were used to analysis genome‐wide expression profile change after SNORD12B knockdown. Microarray analysis of the genome‐wide expression profile was done by KangChen Biotech (Shanghai, China) via Agilent Whole Human genome Oligo Microarray platform. Total RNA was amplified and transcribed into fluorescent cDNA, which was further hybridized onto the Whole Human Genome Oligo Microarray (4 × 44K, Agilent Technologies). After washing in staining dishes, the slides were scanned by the Agilent Scanner G2505C. Differently expressed genes were obtained and the only genes with a fold change of ≥2 and a *p*‐value < .05 were considered.

### 2.18 Immunohistochemistry

Paraffin‐embedded normal brain tissues and glioma tissues were sectioned at 4 μm thickness. The sections were deparaffinized using xylene, rehydrated in graded alcohols (100%, 95%, 80%, and 70%). Then, the sections were incubated with 3% H_2_O_2_ for 10 min and transferred into heated citrate buffer for 30 min for antigen retrieval. After cooling, sections were blocked with 3% BSA/PBS for 15 min and incubated with primary antibody at 4°C for all night. After incubated with secondary antibody Biotinylated Anti‐rabbit/mouse IgG for 30 min at 37°C, the horseradish peroxidase coupled streptavidin was added and incubated with sections for 30 min at 37°C. DAB (3, 3‐diaminobenzidine) was then added and incubated with sections for 20 min at 37°C. After being counterstained with hematoxylin, the sections were dehydrated in graded alcohols, and soaked in xylene. Finally, the sections were mounted and imaged.

### 2.19 Nascent RNA capture

The assay was operated as previously described.^29^ For more details, please see Supporting Information of Materials and Methods.

### 2.20 RNA stability measurement

RNA stability measurement assay was performed as previously described.^29^ Actinomycin D (5 mg/ml) was added to inhibit transcription. For more details, please see Supporting Information of Materials and Methods.

### 2.21 Chromatin immunoprecipitation assay

Chromatin immunoprecipitation (ChIP) assay was operated as previously described.^9^ Immunoprecipitated DNA was amplified with the specific primer by PCR (shown in Table S4). For more details, please see Supporting Information of Materials and Methods.

### 2.22 Tumor xenograft in nude mouse

The constructed stably transfected glioma cells (U251 and U373) were used to establish xenograft models in nude mice. For more details, please see Supporting Information of Materials and Methods.

### 2.23 Gene expression and survival prognosis analysis

We used the “Pathology Atlas” module of the Human Protein Atlas (https://www.proteinatlas.org/) to obtain gene expression data for all TCGA tumors. The “TCGA analysis” module of UALCAN portal (http://ualcan.path.uab.edu/analysis.html) was searched to evaluate gene expression data between glioma and corresponding normal tissues. The “survival analysis” module of GEPIA (http://gepia.cancer‐pku.cn/index.html) was searched to obtain the overall survival plot. For more details, please see Supporting Information of Materials and Methods.

### 2.24 Statistical analysis

The experimental data were collected and presented as mean ± standard deviation (SD). *t*‐Test or one‐way ANOVA was used in the statistical analysis by the GraphPad Prism v8.4 (GraphPad, CA, USA).

## 3 RESULTS

### 3.1 MSI2 was upregulated in GBM tissues and cells, and knockdown of MSI2 inhibited GBM cell glycolipid metabolism and proliferation

**FIGURE 1:**
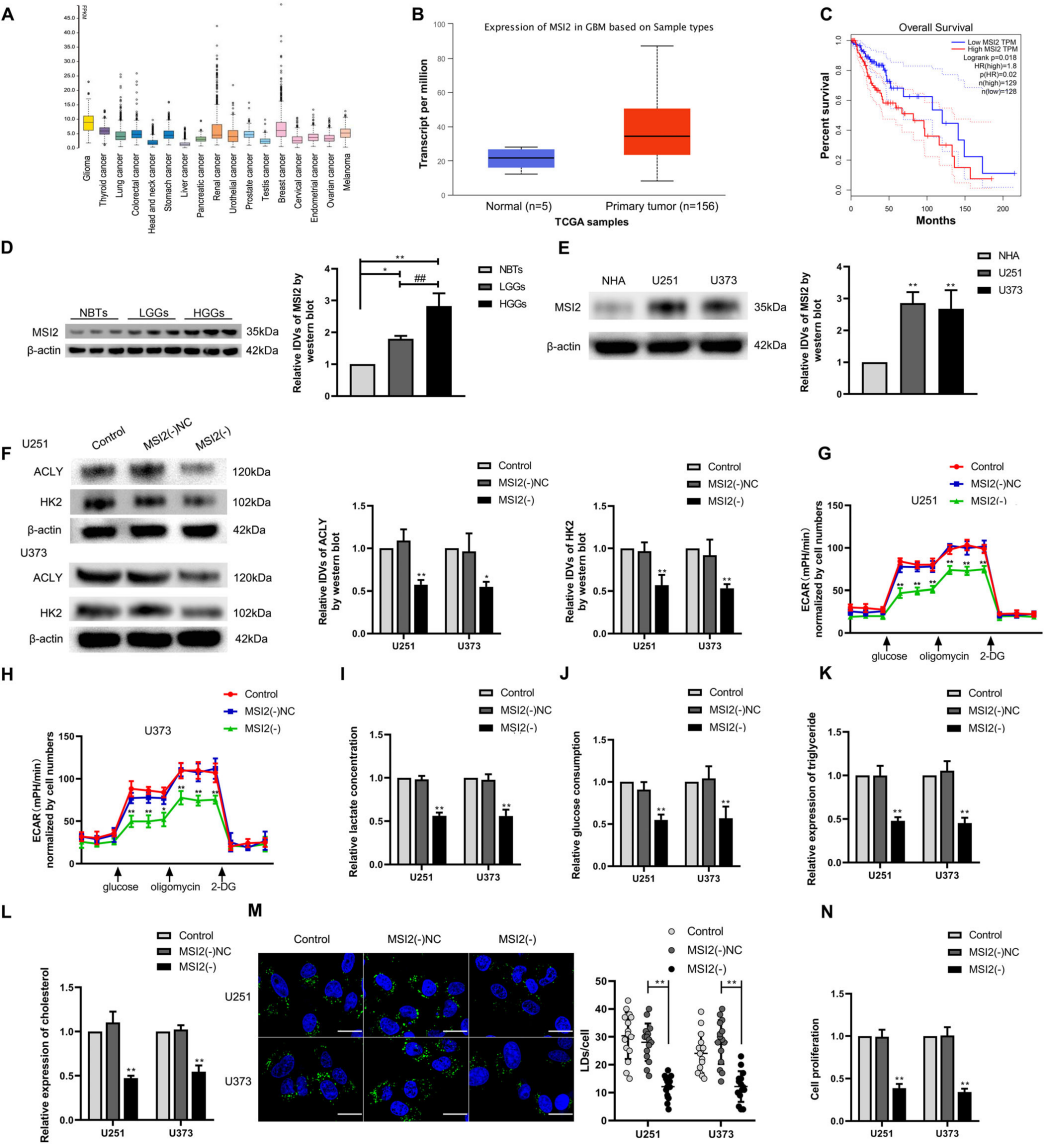
MSI2 expression was elevated in glioma tissues and cells, and knockdown of MSI2 suppressed glycolipid metabolism and proliferation. (A) Expression of MSI2 is high in glioma from TCGA database. (B) Expression of MSI2 in NBTs and glioma from TCGA samples. (C) Effect of MSI2 expression level on LGG patient survival time from TCGA database. (D) Protein level of MSI2 was analyzed in normal brain tissues (NBTs), low‐grade gliomas (LGGs), and high‐grade gliomas (HGGs) via Western blot. ^*^ *p* < .05 versus NBTs group; ^**^ *p* < .01 versus NBTs group; ^##^ *p* < .01 versus LGGs group. (E) MSI2 protein level was analyzed in normal human astrocytes (NHA) and glioma cell lines (U251 and U373) via Western blot. ^**^ *p* < .01 versus NHA group. (F) HK2 and ACLY protein expression after MSI2 knockdown in U251 and U373 cells was analyzed via Western blot. (G and H) The effect of MSI2 knockdown on glycolysis in U251 and U373 cells was analyzed via extracellular acidification rate (ECAR), including glycolysis and glycolytic capacity. (I and J) Lactate production and glucose uptake were measured in U251 and U373 cells after MSI2 knockdown. (K and L) Intracellular triglyceride and cholesterol expression levels were measured after MSI2 knockdown. ^*^ *p* < .05 versus MSI2(−)NC group; ^**^ *p* < .01 versus MSI2(−)NC group. (M) Representative confocal fluorescence imaging of lipid droplets (LDs) stained by BODIPY 493/503 (green) in U251 and U373 cells. Nucleus (blue) was stained by DAPI. Scale bars = 20 μm. Data are presented as the mean ± SD (*n* = 15, each group). ^**^ *p* < .01 versus MSI2(−)NC group. (N) Effect of MSI2 on the proliferation of U251 and U373 cells was detected via Cell Counting Kit‐8 (CCK‐8) assay. ^**^ *p* < .01 versus MSI2(−)NC group. Except for specially noted, data are presented as the mean ± SD of three independent experiments per group. One‐way ANOVA was used for statistical analysis

The expression of MSI2 in glioma was elevated based on data obtained from the Cancer Genome Atlas (Figure 1A,B). Higher expression of MIS2 was related to a shorter survival time in patients with low‐grade glioma (Figure 1C). Immunohistochemical staining showed that MSI2 was mainly expressed in the nucleus of glioma tumor cells and that its expression increased with increasing pathological grade (Figure S1A). Western blot was utilized to explore the expression level of MSI2 protein in normal brain tissues (NBTs), glioma tissues, NHAs, and the GBM cell lines U251 and U373 (Figure 1D,E). As shown in Figure 1D, compared with that in NBTs, the MSI2 protein level in glioma tissues was higher and was increased at higher pathological grades (Figure 1D). Similarly, MSI2 protein expression was significantly upregulated in both U251 and U373 cells compared with that in NHAs (Figure 1E). Based on these results, we further explored the effect of MSI2 on the glycolipid metabolism and proliferation of GBM cells. First, we constructed stable MSI2 knockdown U251 and U373 cell lines (mRNA and protein expression levels for transfection are shown in Figure S2A,B). Western blot revealed that the expression of HK2 and ACLY protein, which are key enzymes in the glycolipid metabolic pathway, decreased significantly when MSI2 was knocked down (Figure 1F). Extracellular acidification rate experiments showed that knockdown of MSI2 significantly reduced aerobic glycolysis in GBM cells (Figure 1G,H). Lactate production and glucose utilization were both significantly reduced after MSI2 knockdown (Figure 1I,J). In terms of lipid metabolism, the levels of intracellular triglyceride and cholesterol were significantly decreased in the MSI2 knockdown group (Figure 1K,L). Additionally, BODIPY 493/503 fluorescence staining revealed that the proportion of lipid droplets was significantly decreased in the MSI2 knockdown group (Figure 1M). The proliferation ability of GBM cells was significantly reduced after MSI2 knockdown according to the results of the Cell Counting Kit‐8 assay (Figure 1N).

### 3.2 SNORD12B was upregulated in GBM tissues and cells, and knockdown of SNORD12B inhibited GBM cell glycolipid metabolism and proliferation

**FIGURE 2:**
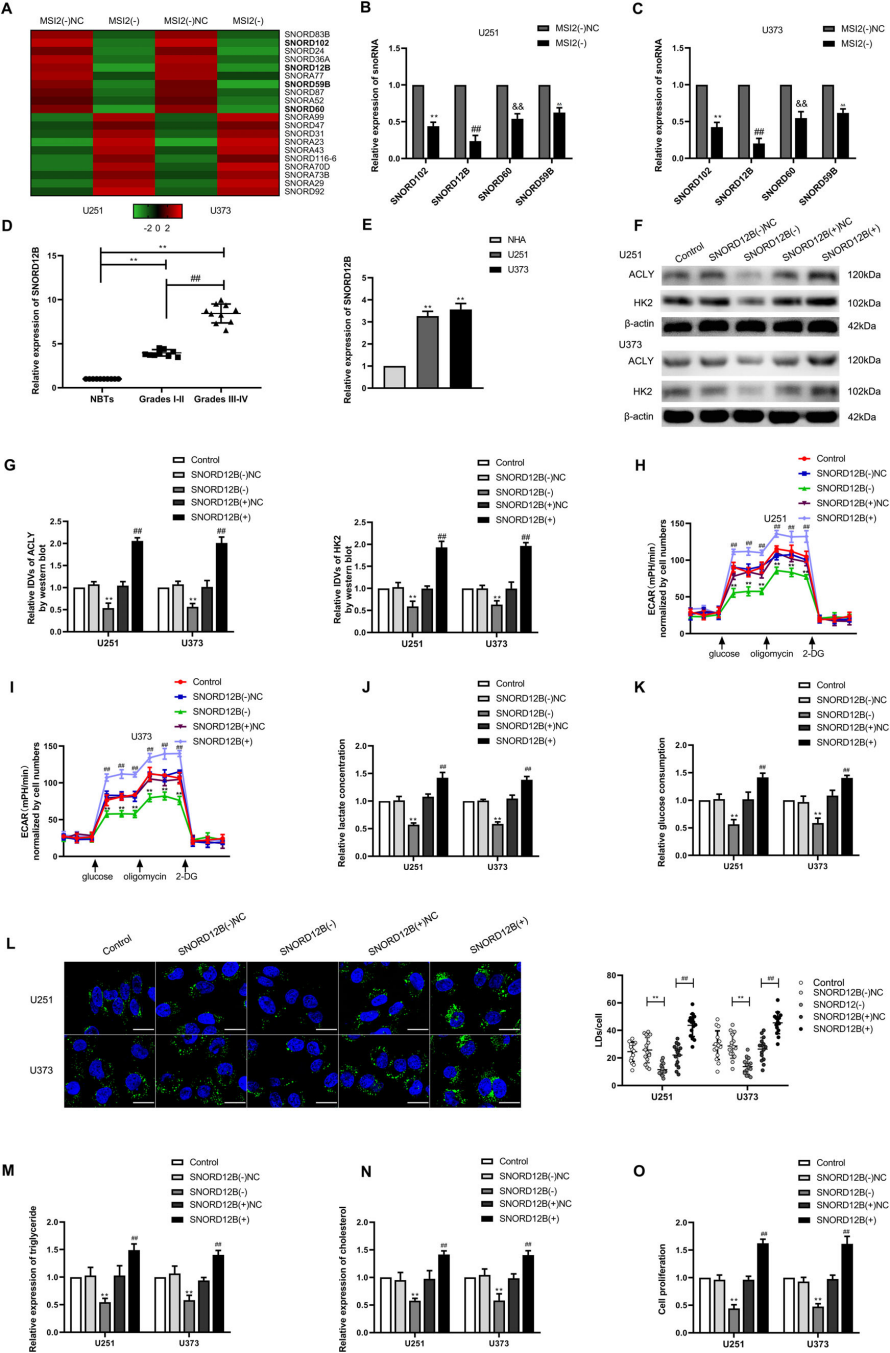
SNORD12B expression was elevated in glioma tissues and cells, and knockdown of SNORD12B suppressed glycolipid metabolism and proliferation. (A) SnoRNA microarray was performed to detect the differential gene when MSI2 was knockdown. (B and C) Selected molecules were validated by qRT‐PCR. ^**/##/&&/ΔΔ^ *p* < .01 versus MSI2(−)NC group. (D) SNORD12B expression was detected in tissues (NBTs [*n* = 10], LGGS [*n* = 10], and HGGs [*n* = 10]) via qRT‐PCR. ^**^ *p* < .01 versus NBTs group; ^##^ *p* < .01 versus LGGs group. (E) SNORD12B expression level was analyzed in NHA cell, U251, and U373 cells via qRT‐PCR. ^**^ *p* < .01 versus NHA group. (F and G) Expressions of HK2 and ACLY were detected via Western blot. (H and I) Effect of SNORD12B on glycolysis was analyzed via ECAR. (J and K) Lactate production and glucose uptake were measured after SNORD12B knockdown or overexpression. ^**^ *p* < .01 versus SNORD12B(−)NC group; ^##^ *p* < .01 versus SNORD12B(+)NC group. (L) Representative confocal fluorescence imaging of LDs stained by BODIPY 493/503 (green) in U251 and U373 cells after SNORD12B knockdown or overexpression. Nucleus (blue) was stained by DAPI. Scale bars = 20 μm. Data are presented as mean ± SD (*n* = 15, each group). ^**^ *p* < .01 versus SNORD12B(−)NC group; ^##^ *p* < .01 versus SNORD12B(+)NC group. (M and N) Intracellular triglyceride and cholesterol expression levels were measured to evaluate the effect of SNORD12B on lipogenesis. (O) Effect of SNORD12B on proliferation was analyzed via CCK‐8. ^**^ *p* < .01 versus SNORD12B(−)NC group; ^##^ *p* < .01 versus SNORD12B(+)NC group. Except for specially noted, data are presented as the mean ± SD of three independent experiments per group. Statistical analysis was by one‐way ANOVA method

The expression profiles of U251 and U373 cells with MSI2 knockdown revealed downregulation of the expression of several snoRNAs based on the results of snoRNA microarray analysis. Several downregulated snoRNAs were validated by qRT‐PCR, with SNORD12B showing the most marked downregulation in expression (Figure 2A–C). We next explored the expression of SNORD12B in different glioma tissues, NHAs, and the U251 and U373 cell lines by qRT‐PCR. The expression of SNORD12B was higher in glioma tissues of different grades compared with that in NBTs, and its expression level was progressively upregulated with increasing pathological grade. Additionally, compared with that in NHAs, SNORD12B expression was significantly elevated in U251 and U373 cells (Figure 2D,E). Further, to analyze the role of SNORD12B in glycolipid metabolism in GBM cells, SNORD12B was overexpressed or knocked down in U251 and U373 GBM cells (gene expression level for transfection is shown in Figure S2C). HK2 and ACLY protein expression levels were significantly downregulated in the SNORD12B knockdown group (Figure 2F,G). Similarly, we observed significant reductions in aerobic glycolysis, lipogenesis, and proliferation following SNORD12B knockdown (Figure 2H–O). However, SNORD12B overexpression increased the protein levels of HK2 and ACLY (Figure 2F,G) and promoted the aerobic glycolytic capacity, lipogenesis, and proliferation of U251 and U373 cells (Figure 2H–O).

### 3.3 MSI2 facilitated glycolipid metabolism of GBM cells by increasing SNORD12B stability

**FIGURE 3:**
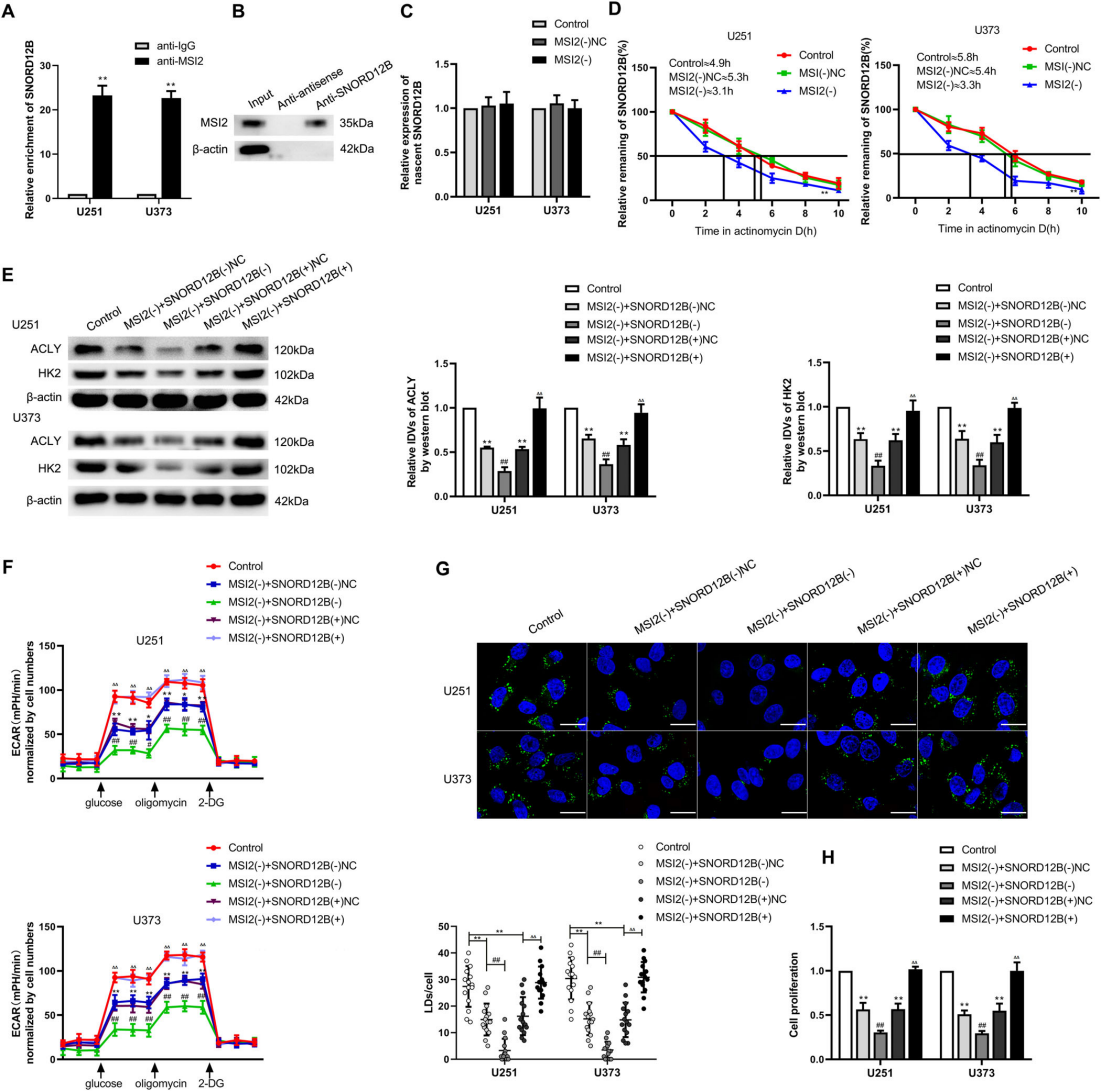
MSI2 facilitated glycolipid metabolism of GBM cells by increasing SNORD12B stability. (A) An enrichment of SNORD12B in MSI2 immunoprecipitated samples via RNA immunoprecipitation (RIP) assay. ^**^ *p* < .01 versus anti‐IgG group, using Student's *t*‐test. (B) RNA pull‐down assay followed by Western blot showed the specific associations of MSI2 with biotinylated‐SNORD12B or antisense RNA. (C) Expression of nascent SNORD12B was measured via qRT‐PCR after MSI2 knockdown. (D) Half‐life of SNORD12B was measured by qRT‐PCR after actinomycin D treated in U251 and U373 cells. ^**^ *p* < .01 versus MSI2(−)NC group. (E) Regulation of HK2 and ACLY expression by MSI2 and SNORD12B was analyzed via Western blot. (F) ECAR was used to measure glycolysis and glycolytic capacity of U251 and U373 cells. ^*^ *p* < .05 versus control group; ^**^ *p* < .01 versus control group; ^#^ *p* < .05 versus MSI2(−) + SNORD12B(−)NC group; ^##^ *p* < .01 versus MSI2(−) + SNORD12B(−)NC group; ^ΔΔ^ *p* < .01 versus MSI2(−) + SNORD12B(+)NC group. (G) Representative confocal fluorescence imaging of LDs stained by BODIPY 493/503 (green) in U251 and U373 cells. Nucleus (blue) was stained by DAPI. Scale bars = 20 μm. Data are presented as the mean ± SD (*n* = 15, each group). ^**^ *p* < .01 versus control group; ^##^ *p* < .01 versus MSI2(−) + SNORD12B(−)NC group; ^ΔΔ^ *p* < .01 versus MSI2(−) + SNORD12B(+)NC group . (H) Effect of MSI2 and SNORD12B on proliferation was analyzed via CCK‐8. ^**^ *p* < .01 versus control group; ^##^ *p* < .01 versus MSI2(−) + SNORD12B(−)NC group; ^ΔΔ^ *p* < .01 versus MSI2(−) + SNORD12B(+)NC group. Except for specially noted, data are presented as the mean ± SD of three independent experiments per group. Statistical analysis was by one‐way ANOVA method

As our results revealed that MSI2 and SNORD12B functioned as oncogenes in GBM cells, and knockdown of MSI2 decreased the expression level of SNORD12B, we further examined the relationship between MSI2 and SNORD12B. According to the bioinformatics database, starBase v2.0, MSI2 was predicted to bind SNORD12B. An RNA immunoprecipitation assay was performed to validate this prediction. As expected, the MSI2‐immunoprecipitated sample showed enrichment of SNORD12B compared with that in the sample in the IgG‐immunoprecipitated group (Figure 3A). Next, binding between MSI2 and SNORD12B was validated by an RNA pulldown assay (Figure 3B). To further clarify the mechanism through which MSI2 upregulated SNORD12B expression in GBM cells, we analyzed the expression of nascent SNORD12B and assessed the half‐life of SNORD12B in MSI2 knockdown cells by qRT‐PCR. As shown in Figure 3C,D, after MSI2 knockdown, the transcripts of nascent SNORD12B exhibited no obvious change, whereas the half‐life of SNORD12B was significantly decreased. We next evaluated whether SNORD12B participated in MSI2 knockdown‐induced inhibition of glycolipid metabolism in GBM cells. SNORD12B(−), SNORD12B(+), and their corresponding NC plasmids were, respectively, transfected into stable MSI2 knockdown cells. Knockdown of SNORD12 strengthened the inhibition of HK2 and ACLY protein expression induced by MSI2 knockdown (Figure 3E). We observed similarly strengthened inhibitory effects on aerobic glycolysis (Figures 3F and S3A,B), lipogenesis (Figures 3G and S3C,D), and cell proliferation (Figure 3H). However, SNORD12B overexpression rescued the suppressive effect on HK2 and ACLY protein expression, glycolipid metabolism, and the proliferation of U251 and U373 cells (Figure 3E–H). These results supported the proposal that SNORD12B is involved in MSI2 knockdown‐induced inhibition of the glycolipid metabolism and proliferation of GBM cells.

### 3.4 ZBTB4 was downregulated in GBM tissues and cells, and overexpression of ZBTB4 inhibited GBM cell glycolipid metabolism and proliferation

**FIGURE 4:**
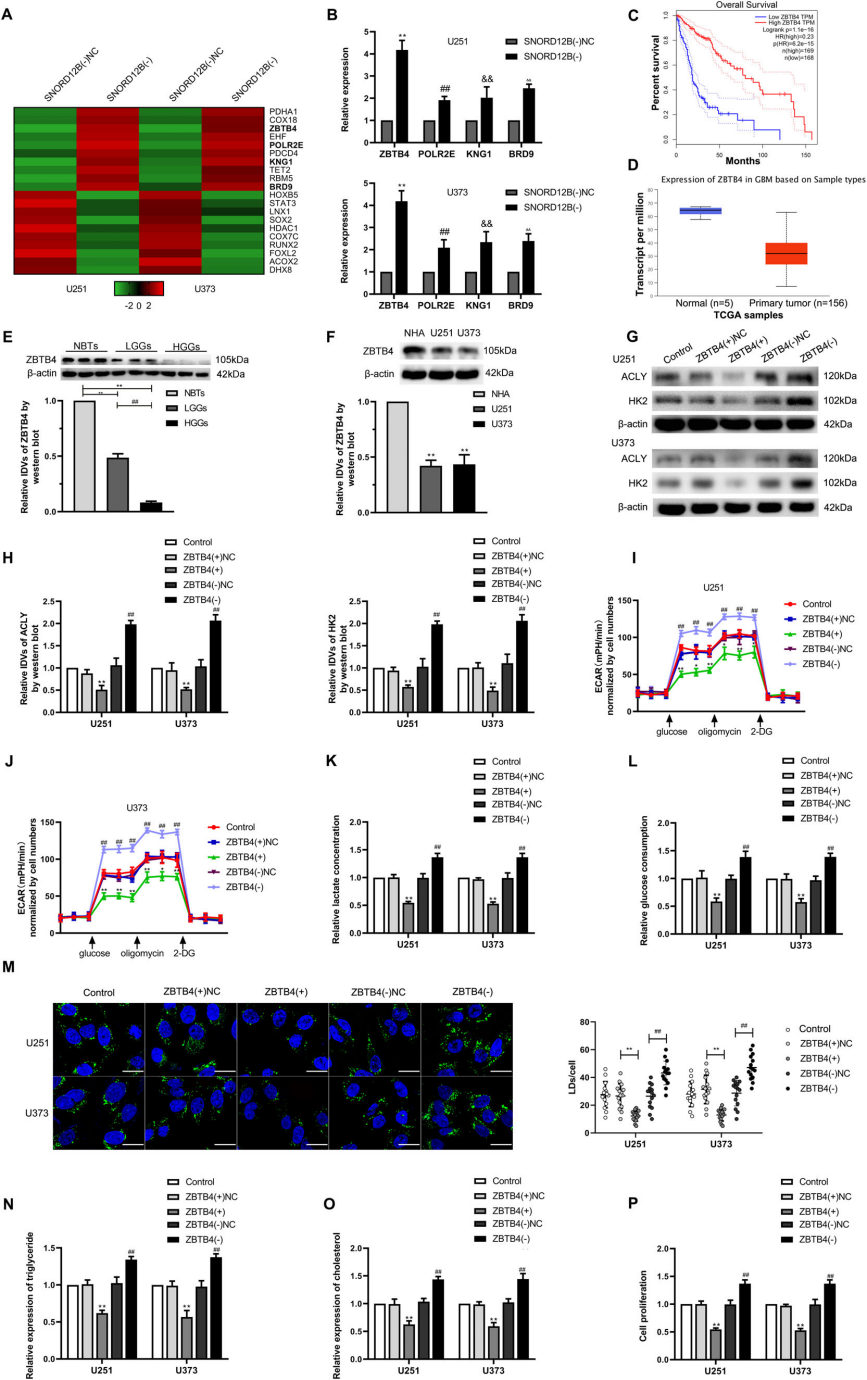
ZBTB4 was downregulated in GBM tissues and cells, and overexpression of ZBTB4 inhibited GBM cells glycolipid metabolism and proliferation. (A) Whole human genome microarray was performed to detect the gene profile when SNORD12B was knockdown. (B) qRT‐PCR was performed to validate the selected molecules. ^**/##/&&/ΔΔ^ *p* < .01 versus SNORD(−)NC group. (C) Effect of ZBTB4 expression level on glioma patient survival time from TCGA database. (D) Expression of ZBTB4 in NBTs and glioma from TCGA samples. (E) ZBTB4 protein levels were analyzed in NBTs, LGGs, and HGGs by Western blot. ^**^ *p* < .01 versus NBTs group; ^##^ *p* < .01 versus LGGs group. (F) ZBTB4 protein levels in NHA, U251, and U373 cells were detected via Western blot. ^**^ *p* < .01 versus NHA group. (G and H) Regulation of HK2 and ACLY expression by ZBTB4 was analyzed via Western blot. (I and J) Effect of ZBTB4 on glycolysis and glycolytic capacity in U251 and U373 cells was measured via ECAR. (K and L) Lactate production and glucose uptake were measured in U251 and U373 cells after ZBTB4 knockdown or overexpression. ^*^ *p* < .05 versus ZBTB4(+)NC group; ^**^ *p* < .01 versus ZBTB4(+)NC group; ^##^ *p* < .01 versus ZBTB4(−)NC group. (M) Representative confocal fluorescence imaging of LDs stained by BODIPY 493/503 (green) in U251 and U373 cells after ZBTB4 knockdown or overexpression. Nucleus (blue) was stained by DAPI. Scale bars = 20 μm. Data are presented as the mean ± SD (*n* = 15, each group). ^**^ *p* < .01 versus ZBTB4(+)NC group; ^##^ *p* < .01 versus ZBTB4(−)NC group. (N and O) Intracellular triglyceride and cholesterol expression levels were measured to evaluate the effect of ZBTB4 on lipogenesis. (P) Effect of ZBTB4 on proliferation was analyzed via CCK‐8 assay. ^**^ *p* < .01 versus ZBTB4(+)NC group; ^##^ *p* < .01 versus ZBTB4(−)NC group. Except for specially noted, data are presented as the mean ± SD of three independent experiments per group. Statistical analysis was by one‐way ANOVA method

The expression profile obtained by whole human genome microarray platform analysis of U251 and U373 cells upon SNORD12B knockdown showed upregulation of several genes. We performed qRT‐PCR to validate these upregulated genes, with ZBTB4 showing the most markedly increased expression in U251 and U373 GBM cells (Figure 4A,B). First, based on the data obtained from The Cancer Genome Atlas, the overall survival of patients with high expression of ZBTB4 was longer than that of patients with low ZBTB4 expression (Figure 4C). ZBTB4 expression was downregulated in gliomas compared with that in NBTs (Figure 4D). Next, we performed Western blot to observe the expression levels of ZBTB4 protein in NBTs, glioma tissues, NHAs, and GBM cells. Compared with that in NBTs, the expression of ZBTB4 protein was significantly lower in different grades of glioma tissues, and the magnitude of the downregulation increased with increasing pathological grade (Figure 4E). Similarly, the expression of ZBTB4 protein was downregulated in U251 and U373 cells compared with that in NHAs (Figure 4F). Further, we analyzed the role of ZBTB4 in glycolipid metabolism in GBM cells. Cells were treated with ZBTB4(+), ZBTB4(−), or their corresponding NC plasmids (mRNA and protein expression levels for transfection are shown in Figure S2D,E). ZBTB4 overexpression induced a significant downregulation in the protein expression levels of HK2 and ACLY (Figure 4G,H) and markedly inhibited the glycolipid metabolism and proliferation of U251 and U373 GBM cells (Figure 4I–P). By contrast, knockdown of ZBTB4 promoted HK2 and ACLY protein expression, glycolipid metabolism, and the proliferation of U251 and U373 GBM cells (Figure 4G–P).

### 3.5 SNORD12B regulated APA of ZBTB4 by competitively binding to FIP1L1

**FIGURE 5:**
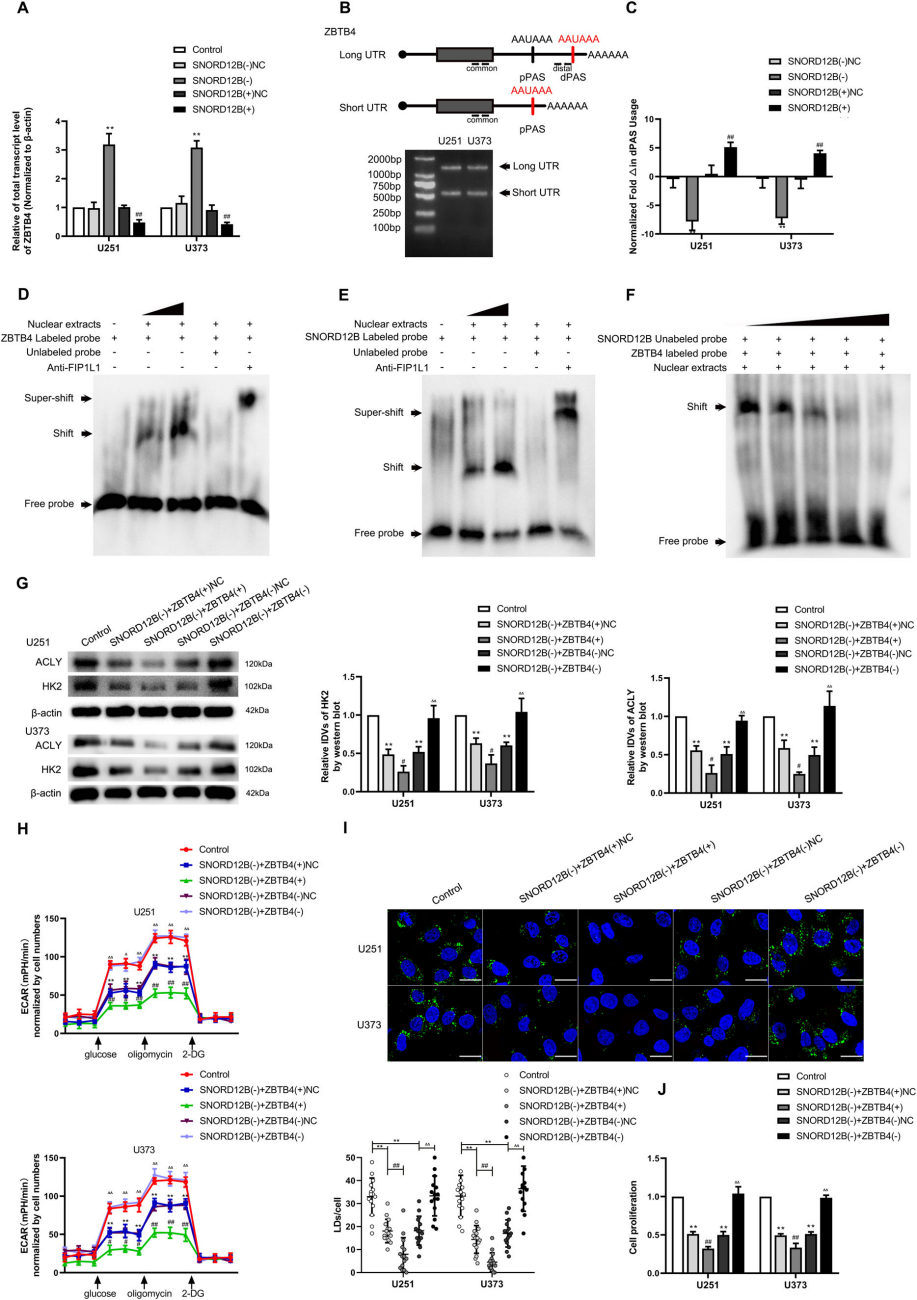
SNORD12B regulated alternative polyadenylation of ZBTB4 by competitively binding to FIP1L1. (A) Effect of SNORD12B on the expression of ZBTB4 mRNA was analyzed by qRT‐PCR. ^**^ *p* < .01 versus SNORD12B(−)NC group; ^##^ *p* < .01 versus SNORD12B(+)NC group by one‐way ANOVA. (B) Diagram showing two polyadenylation signals (PAS) in the 3′UTR of ZBTB4 and the primers specifically designed for the detection of total transcript and long 3′UTR transcript (above). 3′‐Random amplification of cDNA ends (3′RACE) PCR amplify full length 3′UTR of ZBTB4 (below). (C) qRT‐PCR was conducted to analyze the effect of SNORD12B on the utilization of distal polyadenylation signal (dPAS) in U251 and U373 cells. ^**^ *p* < .01 versus SNORD12B(−)NC group; ^##^ *p* < .01 versus SNORD12B(+)NC group by one‐way ANOVA. (D) RNA electrophoretic mobility shift assay (EMSA) showed binding affinity between ZBTB4 and FIP1L1. (E) RNA EMSA showed SNROD12B could bind to FIP1L1. (F) Competitive gel mobility shift assay was performed to detect SNORD12B and ZBTB4 competitively binding to FIP1L1. (G) Regulation of HK2 and ACLY expression by SNORD12B and ZBTB4 was analyzed via Western blot. (H) ECAR to measure glycolysis and glycolytic capacity of U251 and U373 cells regulated by SNORD12B and ZBTB4. **p < .01 versus control group; ^#^ *p* < .05 versus SNORD12B(−) + ZBTB4(+)NC group; ^##^ *p* < .01 versus SNORD12B(−) + ZBTB4(+)NC group; ^Δ^ *p* < .05 versus SNORD12B(−)+ZBTB4(−)NC group; ^ΔΔ^ *p* < .01 versus SNORD12B(−) + ZBTB4(−)NC group. (I) Representative confocal fluorescence imaging of LDs stained by BODIPY 493/503 (green) in U251 and U373 cells. Nucleus (blue) was stained by DAPI. Scale bars = 20 μm. Data are presented as the mean ± SD (*n* = 15, each group). **p < .01 versus control group; ^##^ *p* < .01 versus SNORD12B(−) + ZBTB4(+)NC group; ^ΔΔ^ *p* < .01 versus SNORD12B(−) + ZBTB4(−)NC group. (J) Effect of SNORD12B and ZBTB4 on the proliferation was analyzed via CCK‐8 assay. **p < .01 versus control group; ^##^ *p* < .01 versus SNORD12B(−) + ZBTB4(+)NC group; ^ΔΔ^ *p* < .01 versus SNORD12B(−) + ZBTB4(−)NC group. Except for specially noted, data are presented as the mean ± SD of three independent experiments per group. Statistical analysis was by one‐way ANOVA method

The results of qRT‐PCR revealed that the expression level of ZBTB4 mRNA was significantly increased after SNORD12B knockdown but was decreased when SNORD12B was overexpressed (Figure 5A). Therefore, we further explored the mechanism through which SNORD12B regulated the expression of ZBTB4 in vitro. First, according to bioinformatics analysis using DNAFSMiner, we detected two potential polyadenylation signals (PAS) in the 3′‐untranslated region (UTR) of ZBTB4 mRNA (Figure S4A). We next performed the 3′‐random amplification of cDNA ends PCR to amplify the full‐length 3′‐UTR of ZBTB4. As expected, there were two subtypes, long and short, in the ZBTB4 transcript 3′‐UTR (Figure 5B), which were further confirmed by Sanger sequencing (Figure S4B). As shown in Figure 5B, we designed common primers targeting the open reading frame and presented for total transcripts, and distal primers were designed to target the sequence just before dPAS to detect the long 3′‐UTR transcript that used the dPAS. After knockdown of SNORD12B, dPAS utilization of ZBTB4 was significantly downregulated, demonstrating that the proportion of long 3′‐UTR transcripts decreased in total. By contrast, overexpression of SNORD12B increased the utilization of dPAS of ZBTB4, increasing the proportion of the long 3′‐UTR transcript (Figure 5C). The factor interacting with PAPOLA and CPSF1 (FIP1L1) was a subunit of splicing polyadenylation specific factor (CPSF), which recognized U/A‐rich sequences upstream of the PAS and acted as an important regulator of the APA process. Recent studies showed that U/A‐rich snoRNAs competitively bind to FIP1L1 and regulate APA of the target gene.^30^ SNORD12B is a U/A‐rich snoRNA, and thus, we further explored whether SNORD12B regulated the APA of ZBTB4 via FIP1L1 to regulate ZBTB4 expression. An electrophoretic mobility shift assay showed that both ZBTB4 and SNORD12B can bind to FIP1L1 (Figure 5D,E), and a competitive gel mobility shift assay revealed that SNORD12B competitively binds to FIP1L1 with ZBTB4 (Figure 5F). Together, these results reveal that SNORD12B facilitated dPAS utilization of ZBTB4 in the APA process via FIP1L1 to downregulate the expression of ZBTB4.

To clarify the regulation between SNORD12B and ZBTB4 in the glycolipid metabolism and proliferation of GBM cells, stable SNORD12B knockdown cells were, respectively, treated with ZBTB4(+), ZBTB4(−), or their corresponding NC plasmids. We observed that ZBTB4(+) strengthened the inhibition of protein expression of HK2 and ACLY and reinforced the suppression of glycolipid metabolism, as well as the proliferation induced by SNORD12B knockdown alone (Figure 5G–J and Figure S5A–D). ZBTB4(−) reversed these inhibitory effects.

Western blot assay revealed that ZBTB4 protein levels were markedly increased after MSI2 knockdown (Figure S6A), as well as following SNORD12B knockdown, but were decreased when SNORD12B was overexpressed (Figure S6B). Next, we detected ZBTB4 protein levels in stable MSI2(−) and SNORD12B(−) or SNORD12B(+) cotransfected cells. Cotransfected MSI2(−) and SNORD12B(−) cells showed more significantly increased ZBTB4 protein expression, whereas SNORD12B(+) restored the upregulation of ZBTB4 protein expression induced by MSI2 knockdown alone (Figure S6C). Together, these findings support that MSI2 knockdown increased ZBTB4 protein levels by downregulating SNORD12B expression.

### 3.6 ZBTB4 transcriptionally suppressed the expression of HK2, ACLY, and MSI2 in the manner of binding to their promoter regions directly

**FIGURE 6:**
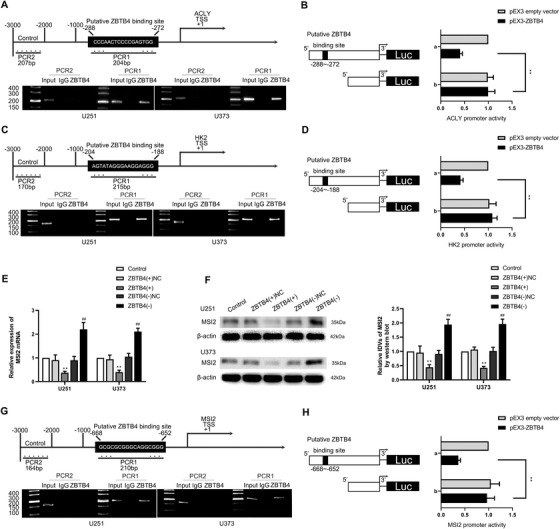
ZBTB4 directly bound to the promoter regions of HK2, ACLY, and MSI2 and transcriptionally suppressed their expression. (A) Putative ZBTB4 binding site is indicated in the ACLY promoter region (above). Chromatin immunoprecipitation (ChIP) assay showed the products amplified putative ZBTB4‐binding sites of ACLY (below). (B) Schematic diagram of luciferase reporter construction and ACLY relative luciferase activity measured in cells cotransfected with the ACLY promoter (−1000 to 0 bp) (or ACLY promoter‐deleted putative ZBTB4 binding site) and pEX3 empty vector or pEX3‐ZBTB4. (C) Putative ZBTB4 binding site is indicated in HK2 promoter region (above). ChIP assay showed the products amplified putative ZBTB4‐binding sites of HK2 (below). (D) Schematic diagram of luciferase reporter construction and HK2 relative luciferase activity measured in cells cotransfected with the HK2 promoter (−1000 to 0 bp) (or HK2 promoter‐deleted putative ZBTB4 binding site) and pEX3 empty vector or pEX3‐ZBTB4. **p < .01 versus pEX3 empty vector group. (E and F) Expression of MSI2 mRNA and protein was measured after ZBTB4 knockdown or overexpression. **p < .01 versus ZBTB4(+)NC group; ^##^ *p* < .01 versus ZBTB4(−)NC group. (G) Putative ZBTB4 binding site was indicated in MSI2 promoter region (above). ChIP assay showed the products amplified putative ZBTB4‐binding sites of MSI2 (below). (H) Schematic diagram of luciferase reporter construction and MSI2 relative luciferase activity measured in cells cotransfected with the MSI2 promoter (−1000 to 0 bp) (or MSI2 promoter‐deleted putative ZBTB4 binding site) and pEX3 empty vector or pEX3‐ZBTB4. **p < .01 versus pEX3 empty vector group. Except for specially noted, data are presented as the mean ± SD of three independent experiments per group. One‐way ANOVA was used for statistical analysis

The expression levels of HK2 and ACLY, including their mRNA expression levels, were significantly decreased following overexpression of ZBTB4 (Figure S7A,B). To further examine the underlying molecular mechanisms, we analyzed the transcription start site for HK2 and ACLY using DBTSS Home. A potential binding site for ZBTB4 was detected in −1000 to 0 bp of HK2 and ACLY promoter regions based on the bioinformatics database Human Transcript Factor Database. Chromatin immunoprecipitation and luciferase reporter assays revealed the interaction between ZBTB4 and the promoter regions of HK2 and ACLY. As shown in Figure 6A–D, the results of these two assays validated that ZBTB4 suppressed the transcription of HK2 and ACLY by directly binding to their respective promoter regions. Therefore, HK2 and ACLY are downstream targets of ZBTB4.

Interestingly, the expression levels of MSI2 mRNA and protein were also elevated by ZBTB4 knockdown but downregulated by ZBTB4 overexpression (Figure 6E,F). Similarly, as shown in Figure 6G,H, ZBTB4 bound to the MSI2 promoter region‐binding site and suppressed MSI2 transcription. Thus, MSI2 regulated ZBTB4 expression via the competitive binding of SNORD12B to FIP1L1, and ZBTB4 transcriptionally suppressed the expression of MSI2, forming a positive feedback loop comprising MSI2/SNORD12B/FIP1L1/ZBTB4.

### 3.7 Simultaneous knockdown of MSI2 and SNORD12B with ZBTB4 overexpression suppressed tumor growth and prolonged survival of nude mice

**FIGURE 7:**
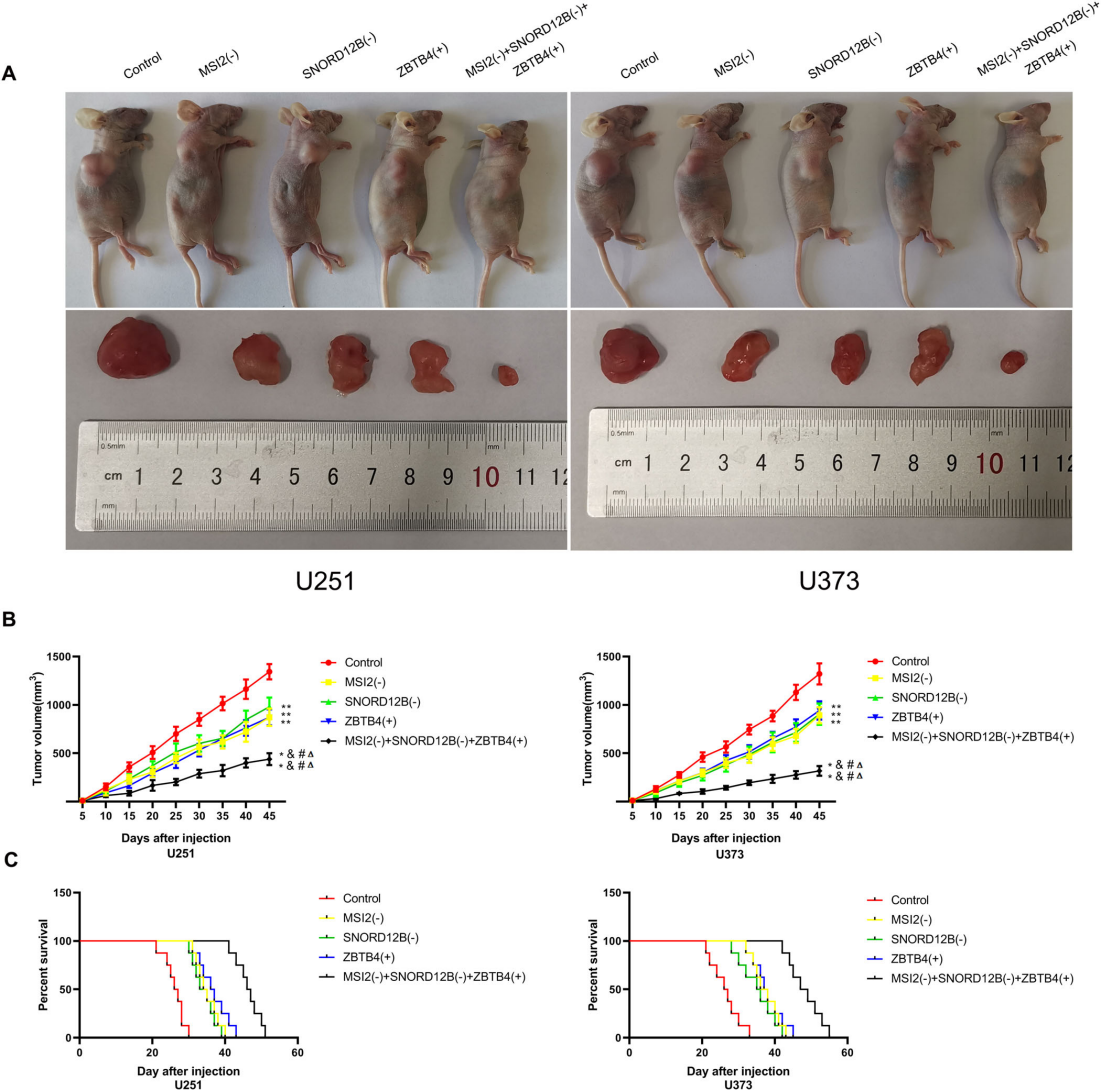
Knockdown of MSI2 and SNORD12B with ZBTB4 overexpression suppressed tumor growth and prolonged survival in nude mice. (A) Subcutaneously xenografted nude mice injected with different treated cells are shown (above). Representative tumors from each group are shown (below). (B) Tumor growth curves are shown. Tumor size was recorded every 5 days, and tumors were extracted at 45 days after injection. **p < .01 versus control group; ^&&^ *p* < .01 versus MSI2(−) group; ^##^ *p* < .01 versus SNORD12B(−) group; ^ΔΔ^ *p* < .01 versus ZBTB4(+) group by one‐way ANOVA. (C) Survival curves of nude mice with orthotopic xenografts are shown. Data are presented as the mean ± SD of eight mice per group

To confirm the roles of MSI2, SNORD12B, and ZBTB4 in tumor growth in vivo, we subcutaneously injected GBM MSI2(−) cells, SNORD12B(−) cells, ZBTB4(+) cells, or a combination of the three cells to construct mouse xenograft models. The average size of the xenograft was smaller in MSI2(−), SNORD12B(−), and ZBTB4(+) mice than that in control mice (Figure 7A,B). The xenograft glioma volume was smallest in the group injected with a combination of the three cells.

**FIGURE 8:**
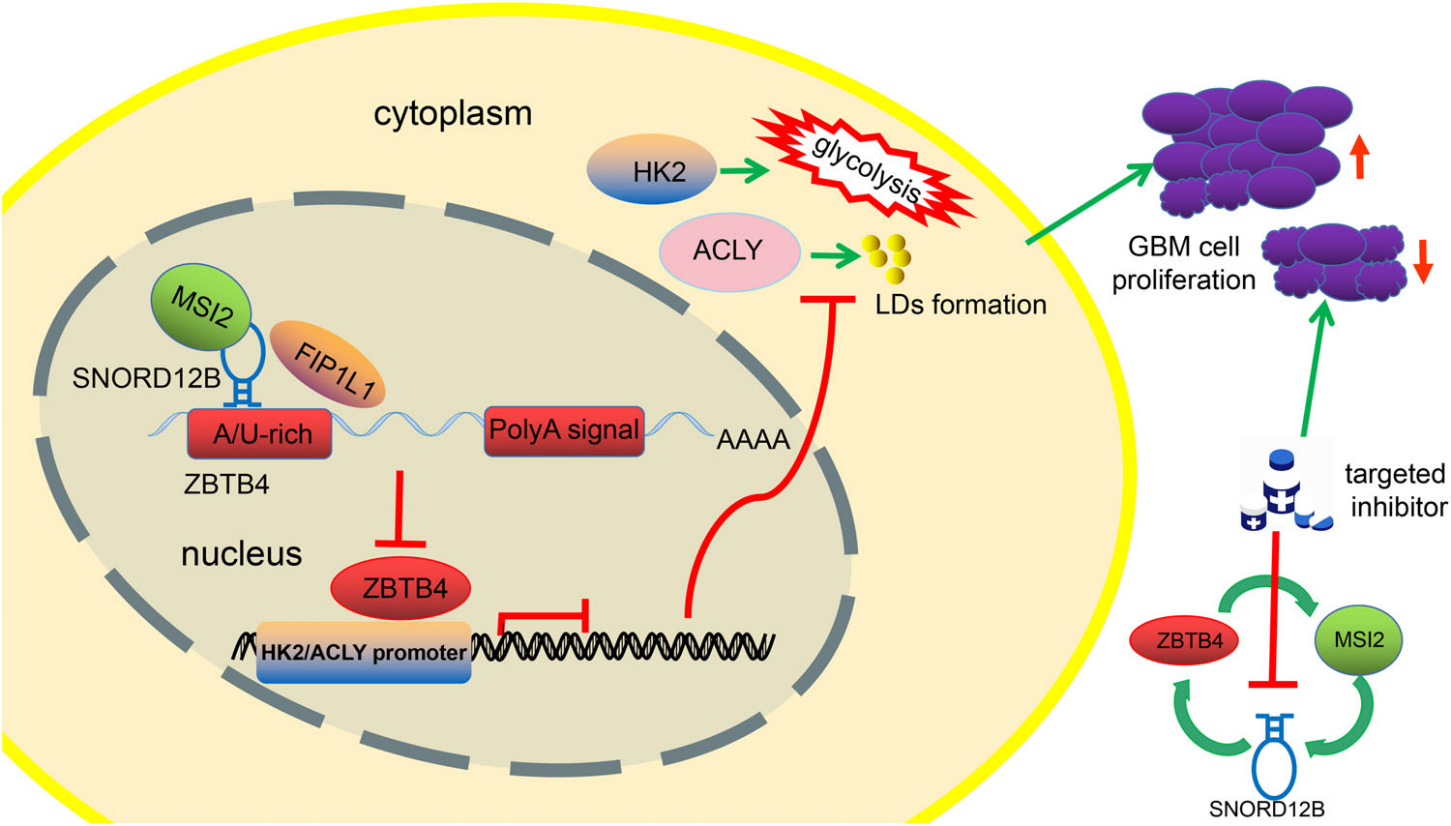
Schematic cartoon of mechanism of MSI2/SNORD12B/FIP1L1/ZBTB4 axis functions as a potential glycolipid metabolism regulator in glioma. MSI2 bound to SNORD12B and upregulated its expression by increasing stability. SNORD12B competitively bound to FIP1L1 with ZBTB4 and facilitated dPAS utilization of ZBTB4 in the APA process, causing downregulation of ZBTB4 expression. ZBTB4 transcriptionally suppressed the expression of HK2, ACLY, and MSI2, forming a positive feedback regulatory loop to collectively regulate the glycolipid metabolism and proliferation of GBM cells

GBM cells were stereotactically implanted into the right striatum of nude mice for survival time analysis. As shown in Figure 7C, mice in the MSI2(−), SNORD12B(−), and ZBTB4(+) groups showed longer survival times than did those in the control group, and mice in the MSI2(−) + SNORD12B(−) + ZBTB4(+) group showed the longest survival time. A schematic diagram of the mechanism underlying the functions of the MSI2/SNORD12B/FIP1L1/ZBTB4 axis as a potential glycolipid metabolism regulator in glioma is shown in Figure 8.

## 4 DISCUSSION

The present study revealed the oncogenic nature of MSI2 and SNORD12B and the antioncogenic role of ZBTB4 in glioma. Knockdown of MSI2 and SNORD12B or overexpression of ZBTB4 significantly inhibited the glycolipid metabolism and proliferation of GBM cells. Knockdown of MSI2 destabilized SNORD12B and decreased its expression level. SNORD12B decreased ZBTB4 expression by facilitating dPAS utilization of ZBTB4 in the APA process. ZBTB4 bind to the HK2 and ACLY promoter regions and inhibit their transcription, thereby impairing glycolipid metabolism and proliferation of glioma cells. We further showed that ZBTB4 inhibited MSI2 mRNA expression at the transcriptional level by binding to its promoter region, thus forming a positive feedback loop consisting of MSI2/SNORD12B/FIP1L1/ZBTB4. To our knowledge, the present study is the first to reveal the value of this loop in regulating the glycolipid metabolism and proliferation of GBM cells.

### 4.1 MSI2, SNORD12B, and ZBTB4 as potential targets for treatment of GBM glycolipid metabolism and proliferation

Accumulating evidence has demonstrated that RBPs are associated with regulating the glycolipid metabolism and proliferation of tumor cells.^9^, ^31^, ^32^ For example, in lung cancer, RBP‐NONO suppresses the proliferation of lung cancer cells by inhibiting the tri‐carboxylic acid cycle and aerobic glycolysis.^33^ RBP‐HuR regulates cellular cholesterol metabolism by mediating ABCA1 gene expression in cancer cells.^34^ In breast cancer, the MSI2 protein level is markedly elevated and promotes the expression of estrogen receptor 1, which facilitates the malignant biological behavior of breast cancer cells.^35^ MSI2 expression is upregulated in cervical cancer cells, and knockdown of MSI2 inhibits the proliferation of cervical cancer cells.^36^ In the present study, a significant increase in MSI2 expression was detected in glioma tissues and GBM cells. MSI2 knockdown significantly inhibited the glycolipid metabolism and proliferation of GBM cells, demonstrating that MSI2 promoted the malignant progression of GBM cells. Recent studies have highlighted the potential role of snoRNAs in regulating tumorigenesis and the energy metabolism of cancer cells. SNORA71A is significantly upregulated in colorectal cancer and liver cancer cells and accelerates the ability of tumor cells in proliferation, migration, and invasion.^37^, ^38^ SNORA37 regulates cancer cell lipid synthesis by mediating histone modification.^39^ SnoRNA Rpl13a is closely related to cell mitochondrial energy metabolism and glucose metabolism.^40^ We found that SNORD12B expression was upregulated in glioma tissues and U251 and U373 cells. SNORD12B knockdown inhibited the glycolipid metabolism and proliferation of GBM cells. In contrast, SNORD12B overexpression promoted the glycolipid metabolism and proliferation of GBM cells. These results suggest that MSI2 and SNORD12B promote the glycolipid metabolism and proliferation of GBM cells and function as oncogenes.

Based on their structural features, snoRNAs are classified as box C/D and box H/ACA snoRNAs, which assemble small nucleolar ribonucleoprotein complexes (snoRNPs) comprising core RNP proteins (NOP58 and NOP56) to facilitate rRNA modification, including uridine isomerization and ribose methylation.^30^, ^41^, ^42^, ^43^ However, accumulating evidence has shown that snoRNAs can assemble into noncanonical snoRNP particles and perform extensive functions to regulate the fate of noncoding RNAs by regulating transcription, splicing, and turnover.^44^, ^45^, ^46^ For example, knockdown of RBP‐PABP can cause defects in snoRNA nuclear processing and disrupt snoRNA homeostasis.^46^ In the current study, we predicted that MSI2 could bind to SNORD12B, which was validated experimentally. More importantly, the expression of SNORD12B was downregulated after MSI2 knockdown, whereas nascent SNORD12B expression was not changed, but the half‐life of SNORD12B was decreased. Therefore, MSI2 may bind to and upregulate the expression of SNORD12B, thereby enhancing its stability and facilitating the glycolipid metabolism and proliferation of GBM cells.

Studies have shown that the expression of ZBTB4 in colon cancer tissues is significantly reduced, and increased ZBTB4 expression significantly prolongs the survival of patients.^47^ ZBTB4 expression in gastric cancer cells is markedly downregulated, and overexpression of ZBTB4 suppresses tumor cell growth.^48^ Similarly, ZBTB4 functions as a tumor suppressor in various tumors. We found that ZBTB4 expression was markedly reduced in GBM tissues and cells, and its expression level decreased with increasing pathological grade. Our results revealed that ZBTB4 inhibited glycolipid metabolism in GBM cells and further inhibited their proliferation.

### 4.2 Alternative polyadenylation regulated by snoRNAs is closely related to tumorigenesis

Next, we examined the mechanism through which SNORD12B regulated the expression of ZBTB4 and further mediated the glycolipid metabolism and proliferation of GBM cells. Recent studies revealed that snoRNAs can regulate gene expression by mediating the APA process of their target gene pre‐mRNAs. For example, SNORD50A, a U/A‐rich snoRNA, can compete with the target pre‐mRNA to bind to FIP1L1 and suppress its APA.^30^, ^49^ FIP1L1 is a subunit of CPSF that recognizes the PAS, which typically binds to U/A‐rich sequences upstream of the PAS and regulates the APA process of target genes.^50^, ^51^ APA allows one gene to produce multiple transcripts with different 3′‐UTR lengths. The 3′‐UTR region of mRNA can bind to transcriptional regulators, such as RBPs, microRNAs, and AU‐rich elements, which have a wide variety of functions, ranging from controlling mRNA stability, translation efficiency, and intracellular localization. Recent studies showed that APA is closely related to tumorigenesis. In proliferating cells, a large number of genes showed enhanced 3′‐UTR shortening via an APA‐mediated mechanism.^52^, ^53^ Transcripts with shortened 3′‐UTRs mainly exhibit markedly increased stability, and gene expression levels are correlated with the loss of AU‐rich elements and miRNA target sites.^54^ In breast cancer, the Ki‐67 gene exhibits extensive 3′‐UTR shortening via APA, and the truncated Ki‐67 mRNA transcript shows improved stability and translation efficiency by avoiding miR‐140‐3p binding and its repressive role.^55^ In the present study, we found that the ZBTB4 mRNA transcript augmented its dPAS usage after SNORD12B overexpression during APA, increasing the proportion of 3′‐UTR long isoforms among mRNA transcripts and decreasing ZBTB4 mRNA and protein expression. Collectively, these findings show that SNORD12B augmented dPAS utilization of ZBTB4 in APA to downregulate the expression of ZBTB4, thus facilitating glycolipid metabolism and proliferation of GBM cells. However, function of the truncated ZBTB4 mRNA transcript requires further analysis.

### 4.3 ZBTB4 acts as an antitumor transcription factor in GBM by regulating glycolipid metabolism pathway

ZBTB4 mainly serves as a transcriptional inhibitor in various cancer cells. In breast cancer cells, ZBTB4 transcriptionally suppresses the expression of EZH2 and inhibits the growth of cancer cells.^19^ Similarly, ZBTB4 can transcriptionally inhibit specific protein transcription factors (Sp1, Sp3, and Sp4), thereby inhibiting the proliferation of prostate cancer cells and promoting cell apoptosis.^20^ We found that ZBTB4 knockdown upregulated the expression of HK2 and ACLY, whereas ZBTB4 overexpression inhibited their expression. Furthermore, the HK2 and ACLY promoter regions were found to possess potential binding sites for ZBTB4. Additionally, ZBTB4 directly bind to the HK2 and ACLY promoter regions and suppress their transcription. Thus, ZBTB4 affected the glycolipid metabolism and proliferation of GBM cells by suppressing the transcription and expression of HK2 and ACLY. Glucose and lipid metabolism are closely related in tumor cells, and ACLY links aberrant glucose and lipid metabolism. ACLY can transport citrate produced by mitochondria during glycolysis to the cytoplasm and catalyze the conversion of citrate into acetyl‐CoA, an important raw material for lipid and cholesterol synthesis in vivo.^56^ Recent studies revealed that ACLY knockdown in tumor cells can cause oxidative stress in tumor cell mitochondria, inhibit cell growth, and promote apoptosis through citrate accumulation.^57^, ^58^ We found that ZBTB4 simultaneously regulated the expression of HK2 and ACLY, the key enzymes involved in glycolipid metabolism. Increased expression of HK2 elevated the level of aerobic glycolysis in GBM cells, leading to the production of large amounts of citrate, acetyl‐CoA, and other macromolecules. Overexpressed ACLY may utilize these compounds to synthesize lipids and cholesterol and store them in lipid droplets to facilitate glycolipid metabolism in GBM cells.

Interestingly, we also confirmed that ZBTB4 could bind to the upstream MSI2 promoter region to transcriptionally repress its expression. A positive feedback loop was formed to regulate the glycolipid metabolism and proliferation of GBM cells. Similar to our results, Zhang et al. found that CDX2 transcriptionally regulates the upstream target gene DGCR8,^29^ and Su et al. reported that SOX3 can bind to the SOX2OT promoter region and transcriptionally activate the expression of SOX2OT, establishing a feedback loop to regulate the malignant behavior of GBM cells.^59^

### 4.4 MSI2–SNORD12B–FIP1L1–ZBTB4 feedback loop as a potential target axis to advance GBM treatment by regulating glycolipid metabolism

Finally, our in vivo study revealed that MSI2 knockdown, SNORD12B knockdown, or ZBTB4 overexpression suppressed xenograft glioma tumor growth and was associated with prolonged survival. Further, mice injected with a combination of the three showed minimal xenograft glioma volume and the longest survival time compared with those in the MSI2 knockdown, SNORD12B knockdown, or ZBTB4 overexpression group. These findings indicate that knockdown of MSI2 and SNORD12B combined with ZBTB4 overexpression has potential clinical value.

## 5 CONCLUSIONS

MSI2 and SNORD12B expression was significantly increased and ZBTB4 expression was decreased in glioma tissues and cells. MSI2 increased SNORD12B stability to upregulate its expression. Overexpressed SNORD12B competitively bound to FIP1L1 with ZBTB4 and facilitated dPAS utilization of ZBTB4 in the APA process, causing downregulation of ZBTB4 expression. ZBTB4 transcriptionally suppressed the expression of HK2, ACLY, and MSI2, forming a positive feedback regulatory loop to collectively regulate the glycolipid metabolism and proliferation of GBM cells. Glioma cells are characterized by metabolic reprogramming, including enhanced aerobic glycolysis and lipid synthesis. Metabolite profiling of cerebrospinal fluid of glioma patients can reveal glioma grade and leptomeningeal metastasis.^60^ Inhibitors of key enzymes and transcription factors involved in glycolipid metabolism have been evaluated in preclinical trials and are considered novel targets for cancer treatment.^10^ Although therapies targeting tumor metabolic reprogramming can kill tumor cells, the associated toxic effects have restricted their application. Most targeted therapy drugs found therapeutically effective and safe in animal models failed in clinical trials due to toxic effects.^61^ Compared with previous cancer metabolism studies that focused on either the glycolytic pathway or the lipid synthesis pathway, the feedback loop investigated in this study simultaneously regulates two biosynthetic metabolic pathways. In the context of translational medicine, the molecular target identified in this loop may offer better value for glioma treatment, as inhibitors at lower or tolerable doses may be sufficient to suppress tumor progression based on a combination of the inhibitory effects on both glycolysis and lipid synthesis. In the context of predictive, preventive, and personalized medicine, a deeper understanding of the reprogramming of energy metabolism in glioma cells can contribute to improving anticancer treatment and the establishment of individualized therapeutic targets for each patient.

## CONFLICT OF INTEREST

The authors declare that there is no conflict of interest.

## AUTHOR CONTRIBUTIONS

Yixue Xue and Yunhui Liu designed the study. Weiwei Dong, Xiaobai Liu, Chunqing Yang, Di Wang, Xuelei Ruan, Mengyang Zhang, and Jian Song performed the experiments and acquired the data. Heng Cai, Jian Zheng, Weiwei Dong, Xiaobai Liu, Chunqing Yang, Mengyang Zhang, and Jian Song analyzed the data. Weiwei Dong and Xiaobai Liu contributed to discussion and writing of the manuscript.

## Supporting information

Figure S1

Figure S2

Figure S3

Figure S4

Figure S5

Figure S6

Figure S7

Figure S8

Supporting Information

Table S1 The primers of MSI2 mRNA, ZBTB4 mRNA, HK2 mRNA, ACLY mRNA, POLR2E mRNA, KNG1 mRNA, and BRD9 mRNA in quantitative real‐time PCR (qRT‐PCR)
Table S2 Sequences of sgRNA for knockdown of SNORD12B
TableS3 The short hairpin RNAs against MSI2 and ZBTB4 sites
Table S4 Primers used for ChIP experiments

## Data Availability

The data that support the findings of this study are available from the corresponding author upon reasonable request.
