# Supplementary figures and images for: Glioma glycolipid metabolism: MSI2–SNORD12B–FIP1L1–ZBTB4 feedback loop as a potential treatment target
Source: Clin Transl Med. 2021 May 12;11(5):e411. doi: 10.1002/ctm2.411 (PMC8114150; doi:10.1002/ctm2.411)

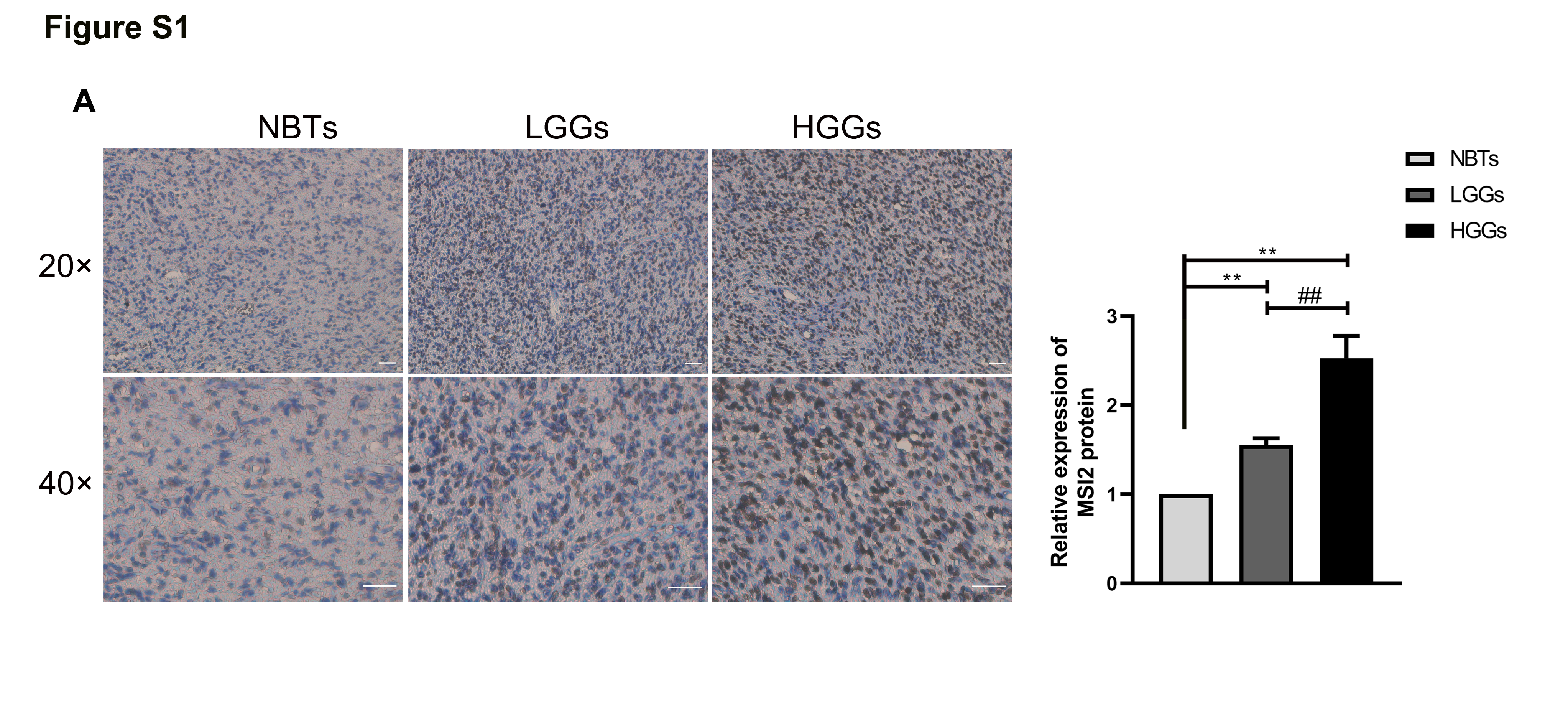

Supplement: Supplementary file 1 — Figure S1 [file CTM2-11-e411-s003.tif]

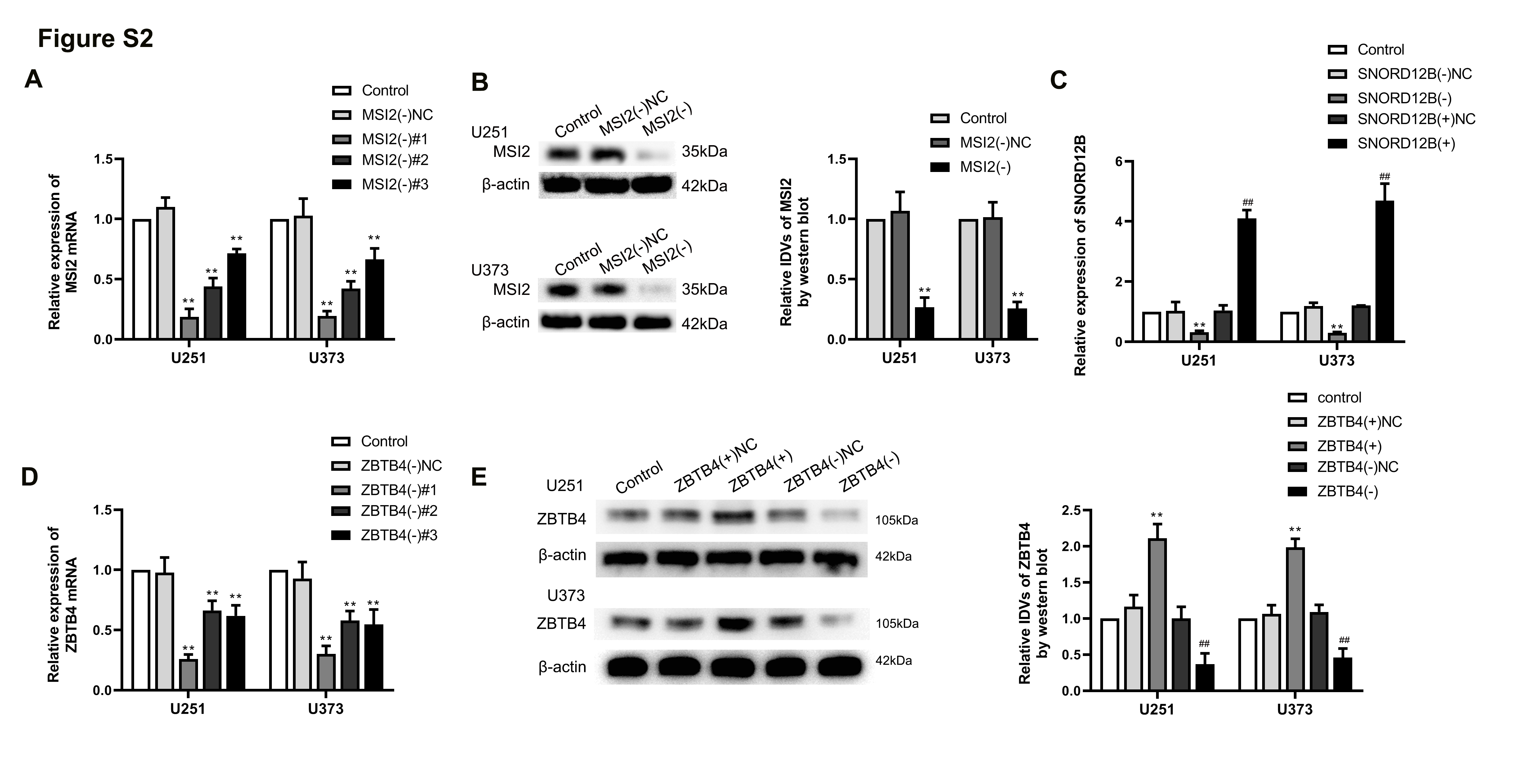

Supplement: Supplementary file 2 — Figure S2 [file CTM2-11-e411-s007.tif]

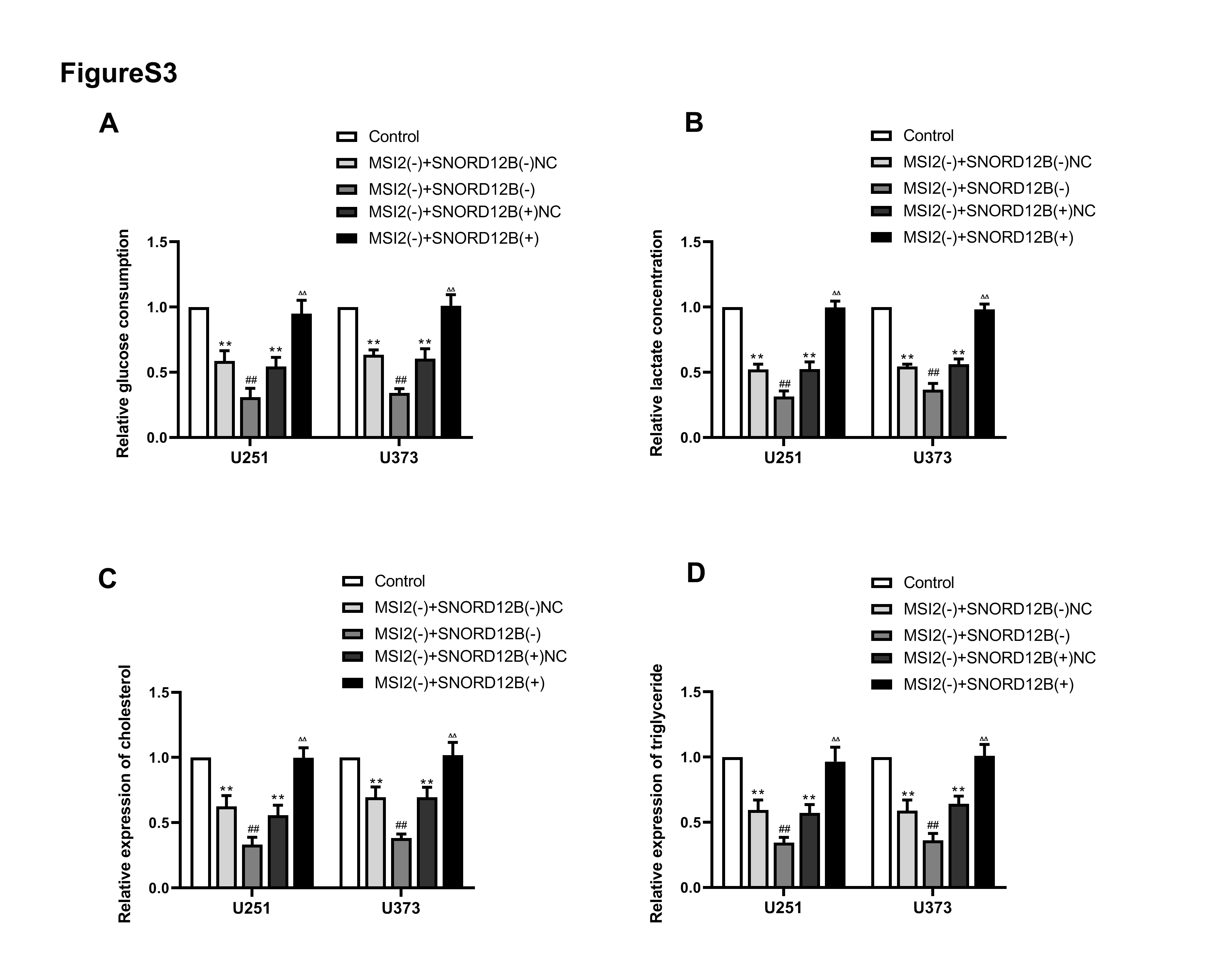

Supplement: Supplementary file 3 — Figure S3 [file CTM2-11-e411-s004.tif]

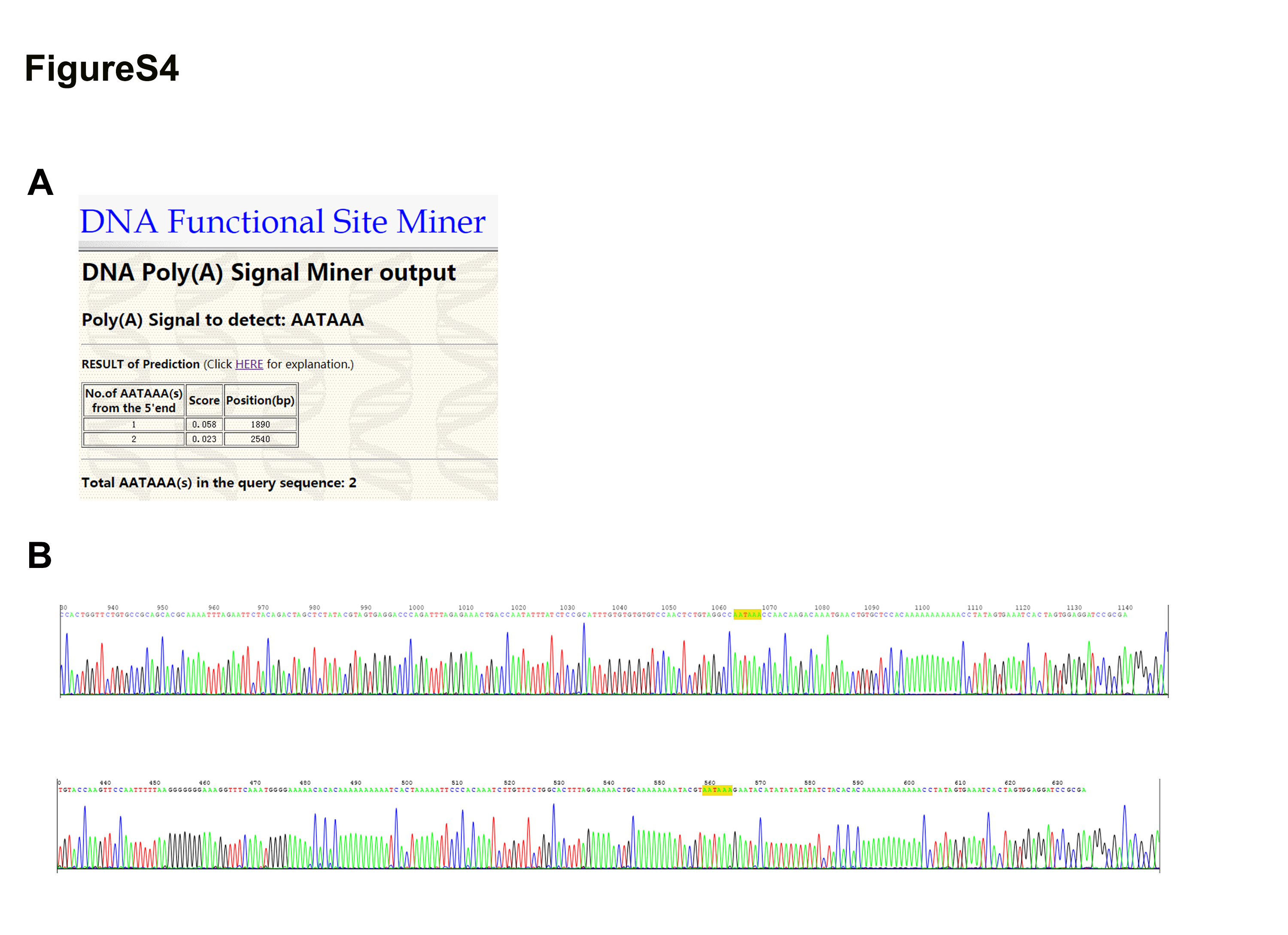

Supplement: Supplementary file 4 — Figure S4 [file CTM2-11-e411-s002.tif]

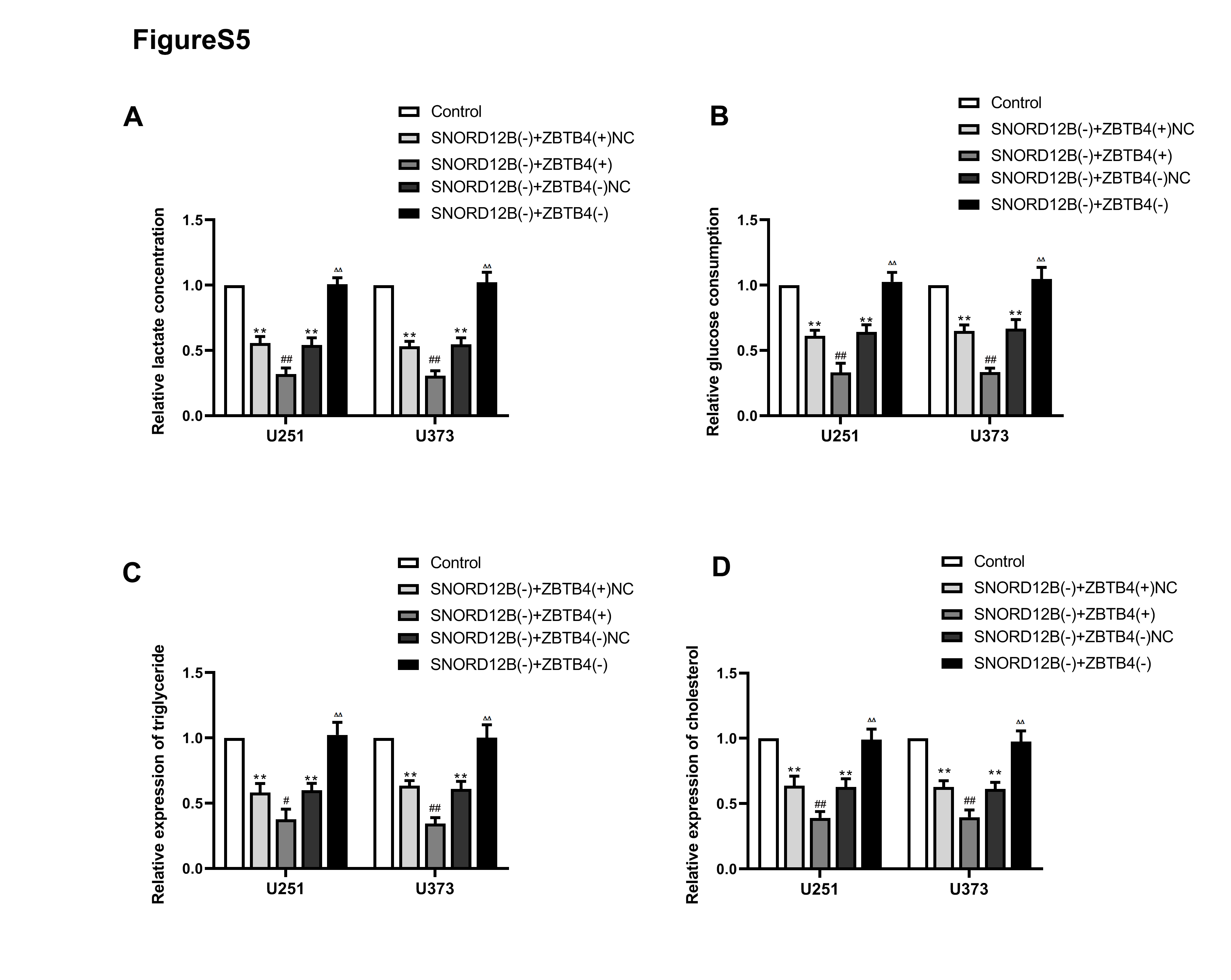

Supplement: Supplementary file 5 — Figure S5 [file CTM2-11-e411-s005.tif]

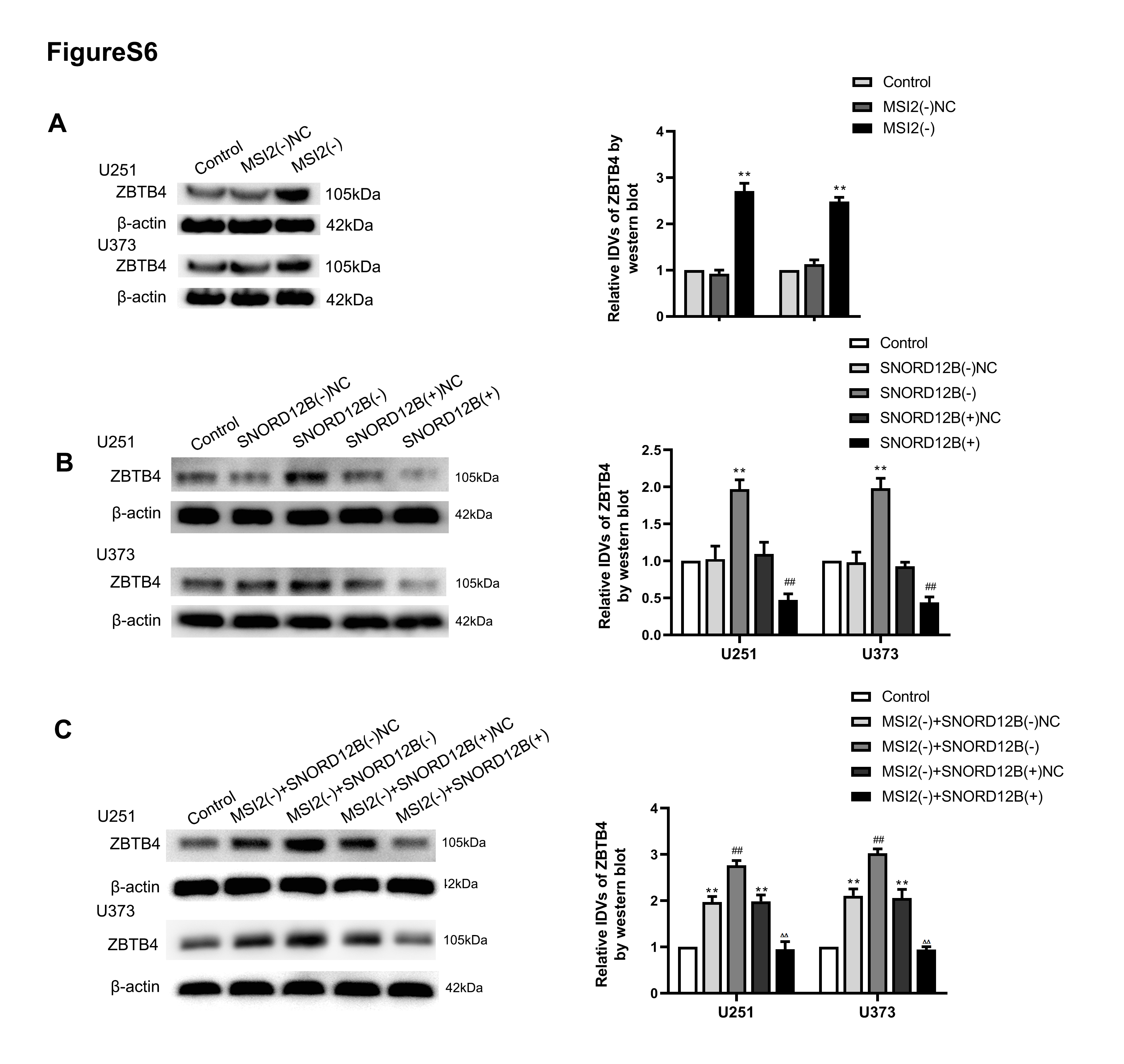

Supplement: Supplementary file 6 — Figure S6 [file CTM2-11-e411-s006.tif]

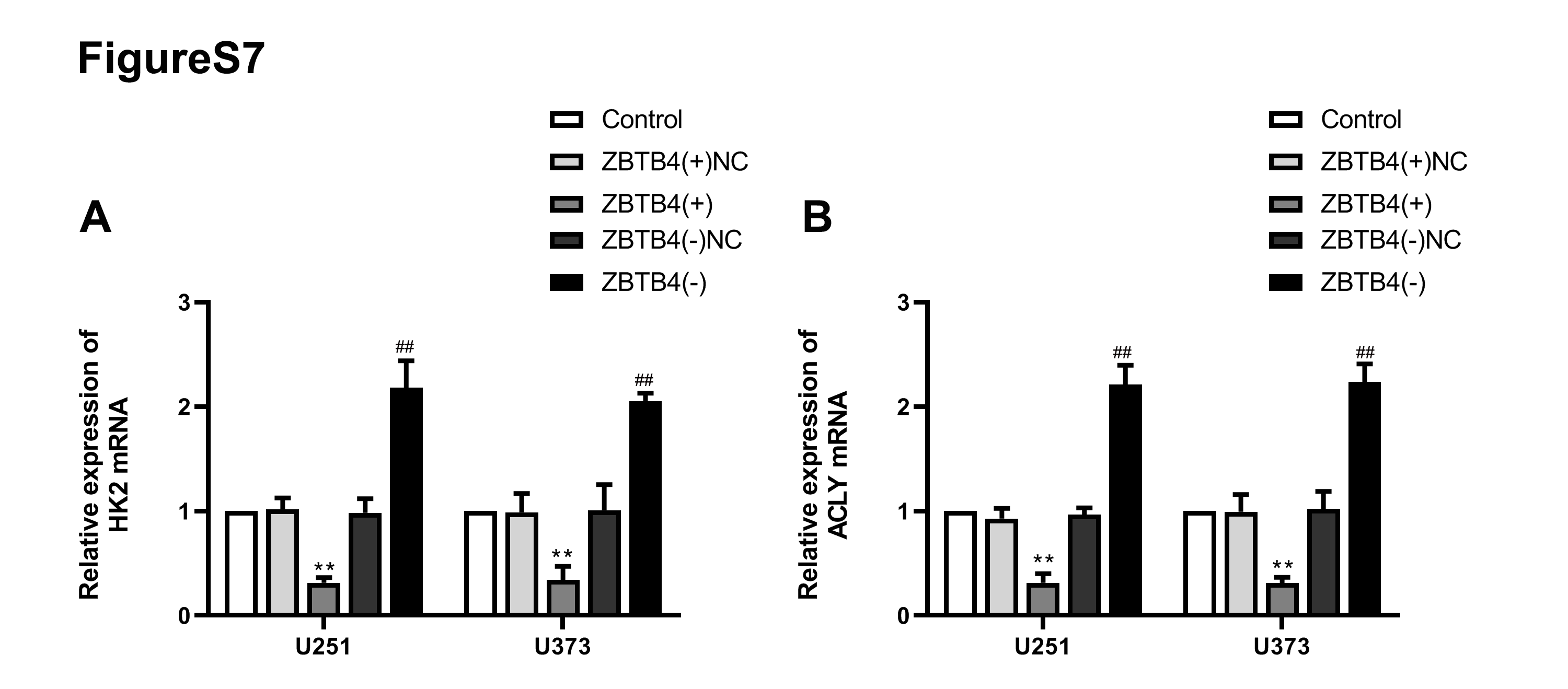

Supplement: Supplementary file 7 — Figure S7 [file CTM2-11-e411-s008.tif]
