# Supplementary material for: Glioma glycolipid metabolism: MSI2–SNORD12B–FIP1L1–ZBTB4 feedback loop as a potential treatment target
Source: Clin Transl Med. 2021 May 12;11(5):e411. doi: 10.1002/ctm2.411 (PMC8114150; doi:10.1002/ctm2.411)
Supplement: Supplementary file 8 — Figure S8 [file CTM2-11-e411-s001.doc]

**Supporting information Figure S1**

**Selected MSI2 as candidates**

1. Immunohistochemistry of MSI2 protein expression in normal brain tissues (NBTs), low grade gliomas (LGGs), and high grade gliomas (HGGs). Scale bars = 50µm. ^**^p < .01 versus NBTs group; ^##^ p < .01 versus LGGs group. Data are presented as the mean ± SD of three independent experiments per group.

**Supporting information Figure S2**

**Transfection efficiency of MSI2, SNORD12B, ZBTB4**

1. Efficiency of transient knockdown of MSI2 three different sites. (B) Efficiency of stable transfection of MSI2 site #1. **p < .01 versus MSI(-)NC group. (C) Efficiency of stable transfection of SNORD12B overexpression or knockdown. **p < .01 versus SNORD12B(-)NC group; ^##^p < .01 versus SNORD12B(+)NC group. (D) Efficiency of transient knockdown of ZBTB4 three different sites. **p < .01 versus ZBTB4(-)NC group (E) Efficiency of table transfection of ZBTB4 overexpression or knockdown site #1. **p < .01 versus ZBTB4(+)NC group; ^##^p < .01 versus ZBTB(-)NC group. Data are presented as the mean ± SD of three independent experiments per group. One-way ANOVA was used for statistical analysis.

**Supporting information Figure S3**

**Effect of MSI2 and SNORD12B on glycolipid metabolism in U251 and U373 cells**

(A, B) Effect of MSI2 and SNORD12B on glucose consumption and lactate production were analyzed in U251 and U373 cells. (C, D) Effect of MSI2 and SNORD12B on the intracellular cholesterol and triglyceride expression level were analyzed. ^**^p < .01 versus control group; ^##^p < .01 versus MSI2(-)+SNORD12B(-)NC group; ^ΔΔ^p < .01 versus MSI2(-)+SNORD12B(+)NC group. Data are presented as the mean ± SD of three independent experiments per group. One-way ANOVA was used for statistical analysis.

**Supporting information Figure S4**

**There were two polyadenylation signals (PAS) in the 3’UTR of ZBTB4 mRNA**

1. Bioinformatics DNAFSMiner database predicted two PAS (AATAAA) in the 3’UTR of ZBTB4. (B) Sanger sequencing of the long and short 3’UTR sequence of ZBTB4.

**Supporting information Figure S5**

**Effect of SNORD12B and ZBTB4 on glycolipid metabolism in U251 and U373 cells**

(A, B) Effect of SNORD12B and ZBTB4 on glucose consumption and lactate production were analyzed in U251 and U373 cells. (C, D) Effect of SNORD12B and ZBTB4 on the intracellular triglyceride and cholesterol expression level were analyzed. **p < .01 versus control group; ^#^p < .05 versus SNORD12B(-)+ZBTB4(+)NC group; ^##^p < .01 versus SNORD12B(-)+ZBTB4(+)NC group; ^ΔΔ^p < .01 versus SNORD12B(-)+ZBTB4(-)NC group. Data are presented as the mean ± SD of three independent experiments per group. One-way ANOVA was used for statistical analysis.

**Supporting information Figure S6**

**Effect of MSI2 and SNORD12B on the protein expression of ZBTB4**

(A) Western blot to detect ZBTB4 protein expression after MSI2 knockdown. ^**^p < .01 versus MSI2(-)NC group. (B)The effect of SNORD12B on the protein expression of ZBTB4. ^**^p < .01 versus SNORD(-)NC group; ^##^p < .01 versus SNORD(+)NC group. (C) The effect of MSI2 and SNORD12B on the protein expression of ZBTB4. **p < .01 versus control group; ^##^p < .01 versus MSI2(-)+SNORD12B(-)NC group; ^ΔΔ^p < .01 versus MSI2(-)+SNORD12B(+)NC group. Data are presented as the mean ± SD of three independent experiments per group. One-way ANOVA was used for statistical analysis.

**Supporting information Figure S7**

**ZBTB4 transcriptionally suppressed the mRNA expression of HK2 and ACLY**

1. The mRNA expression level of HK2 after ZBTB4 overexpression and knockdown. (B) The mRNA expression level of ACLY after ZBTB4 overexpression and knockdown. **p < .01 versus ZBTB4(+)NC group; ^##^p < .01 versus ZBTB(-)NC group. Data are presented as the mean ± SD of three independent experiments per group. One-way ANOVA was used for statistical analysis.
