# Supplementary material for: Glioma glycolipid metabolism: MSI2–SNORD12B–FIP1L1–ZBTB4 feedback loop as a potential treatment target
Source: Clin Transl Med. 2021 May 12;11(5):e411. doi: 10.1002/ctm2.411 (PMC8114150; doi:10.1002/ctm2.411)
Supplement: Supplementary file 9 — Supporting Information [file CTM2-11-e411-s010.doc]

**Supporting information materials and methods**

**Clinical Tissues**

Normal brain tissues (n=10) and glioma tissues were obtained from the Department of Neurosurgery, Shengjing Hospital of China Medical University. The glioma tissues were classified into low grade glioma (LGGs, WHO grade I-II, n=10) and high grade glioma (HGGs, WHO grade III-IV, n=10), according to the 2007 World Health Organization classification. NBTs were collected from donation of individuals who died in traffic accident and free of any prior pathologically detectable condition. All the tissue samples were immediately frozen in liquid nitrogen after surgical resection and stored in liquid nitrogen until use.

**Cell Culture**

U251 and U373 tumor cells were cultured in Dulbecco’s modified Eagle’s medium (DMEM, HyClone, USA) supplemented with 10% fetal bovine serum (FBS, Gibco, Grand Island, NY, USA). NHA cells were cultured in RPMI-1640 medium (Gibco, Grand Island, NY, USA) with 10% FBS. All cells were maintained in a humidified incubator at 37℃ with 5% CO_2_.

**RNA extraction and quantitative real-time PCR**

After total RNA was extracted from cells and tissues with Trizol reagent, RNA concentration and quality were determined via 260/280nm absorbance with Nanodrop Spectrophotometer (ND-100, Thermo, USA). One-step SYBR PrimeScript RT-PCR Kit (TakaraBio, Inc., Japan) was used to detect the expression of target gene mRNA via 7500 Fast real-time PCR system (Applied Biosystems, USA). β-actin was used as endogenous control. The snoRNA expression was detected by using Bulge-Loop^TM^ miRNA qRT-PCR system (Ribobio, Guangzhou, China) according to the manufacture’s instructions. Primers of snoRNAs (SNORD12B, SNORD60, SNORD102, SNORD59B and U6) were designed by Ribobio Corporation (Guangzhou, China). U6 was used as endogenous control. Three independent experiments were performed for each sample. Melting curve analysis was performed at the end of experiment to validate the specificity of the expected PCR product. The expression levels were normalized to the endogenous controls and calculated as fold change using the relative quantification(2^-∆∆Ct^) method.

**Cell transfection**

Cells were seeded in a 24-well plates and transfected with plasmids when cells cultured to 70-80% confluence using Lipofectamine 3000 reagent (Life Technologies, Carlsbad, CA, USA) following manufacture’s protocol. G418, puromcin, and blastmycin (Sigma-Aldrich, StLouis, MO, USA) were used to selected the stable transfected cells. Resistant cell clones were established around approximately 4 weeks.

**Western blotting**

Total proteins were lysed with RIPA buffer with protease inhibitors (Beyotime Institute of Biotechnology, Jiangsu, China). The protein concentration was analyzed by BCA protein assay (Beyotime Institute of Biotechnology, Jiangsu, China). Equal amount of protein sample was subjected to SDS/PAGE gels and transferred to PVDF membranes. Membranes were blocked with 5% nonfat milk in tris-buffered saline (TBS) containing 0.1% Tween-20 (TBST) for 2h at room temperature and then were incubated with primary antibodies overnight at 4℃as follows: MSI2 (1:3000; Proteintech, Chicago, IL, USA), ZBTB4 (1:500; Santa Cruz Biotechnology, CA, USA), HK2 (1:5000; Proteintech, Chicago, IL, USA), ACLY(1:500; Santa Cruz Biotechnology, CA, USA), β-actin (1:5000; Proteintech, Chicago, IL, USA). After washing with TBST for 3 times, the membranes were incubated with respective appropriate horseradish peroxidase conjugated secondary antibodies at room temperature for 2h as follows: goat anti-mouse (1:10,000; Proteintech, Chicago, IL, USA) or goat anti-rabbit (1:10,000; Proteintech, Chicago, IL, USA). The blots were visualized with enhanced chemiluminescence (ECL) kit (Beyotime Institute of Biotechnology, Jiangsu, China) and scanned by ChemImager 5500 V2.03 software. The relative integrated density values (IDV) were calculated using Image-J software based on β-actin as endogenous control.

**Measurement of extra cellular acidification rate**

The ECAR was examined with XF glycolysis stress test kit (Seahorse Bioscience, USA) according to the instructions of manufacturer. Briefly, 5×10^5^ cells per well were plated into Seahorse plates, maintained in 500ul meduim overnight at 37℃ under 5%CO2. Next day, the cells were cultured in XF Base Medium and incubated at 37℃ in a non-CO2 incubator to maintain glucose starvation condition. Seahorse buffer including glucose (final concentration 10nM), oligomycin and 2-deoxyglucose (2-DG) were injected into the XF24 Extracellular Flux Analyzer (Seahorse Bioscience, Billerica, MA, USA) in the proper order to measure ECAR. After measurement, cell number was rechecked and measurements were normalized by cell numbers.

**Dual-luciferase reporter assay**

The wild type vector containing promoter region sequences of HK2, ACLY and MSI2 and mutant type vector truncated sequences were amplified by PCR respectively. The PCR products were subcloned into the pGL3-Basic vector (Promega, WI, USA) to obtain different recombinant vectors. We also constructed the pEX3-ZBTB4 recombinant vector (GenePharma, Shanghai, China) containing human full-length ZBTB4 sequence. The HEK-293T cells were co-transfected with the recombinant wild type or mutant luciferase expression vector and pEX3-ZBTB4 or empty vector. The luciferase activity was detected at 48h after co-transfection using Dual-Luciferase reporter assay kit (Promega, WI, USA) following the manufacturer’s protocol.

**RNA immunoprecipitation (RIP) assay**

RIP assay was performed using an EZ-Magna RNA-binding protein immunoprecipitation kit (Millipore,USA) according to the manufacture’s protocol. A MSI2 antibody (Proteintech, Chicago, IL, USA) was used for the RIP assay and IgG was used as a negative control. The cell lysate was incubated with compounds of RIP buffer, magnetic beads, and antibody. Then, the compound were incubated with proteinase K, and immunoprecipitated RNA was isolated. The RNA concentration was measured by Nanodrop Spectrophotometer (ND-100, Thermo, USA). Lastly, the purified RNA was analyzed by qRT-PCR to demonstrate the presence of the binding targets.

**RNA pull-down assay**

The interaction between MSI2 and SNORD12B was detected using Pierce Magnetic RNA-Protein Pull-down Kit (Thermo Fisher, Carlsbad, CA, USA) according to the manufacture’s instruction. In brief, biotin-labeled SNORD12B or antisense RNA was synthesized, and co-incubated with the cell lysates for 4h. Then, the protein combined with biotin-labeled SNORD12B or antisense RNA was pull down with sreptavidin magnetic beads after incubation overnight. The bead-RNA-protein mixture was collected by low-speed centrifuge, and eluted through Handee spin columns. Wash the bead-RNA-protein mixture with the boiled SDS buffer. The retrieved proteins were detected by western blot with β-actin as the control.

**Cell viability assay**

Cell Counting kit-8 (CCK-8) assay (Beyotime Institute of Biotechnology, Jiangsu, China) was performed to determine the viability of glioma cell. 2×10^3^ cells were seeded in 96-well plates. Each group had 3 replicates. After 48h incubation, 10ul CCK-8 solution was added into each well and incubated with cells for 2h. Absorbance was measured at a wavelength of 450nm using SpectraMax M5 microplate reader (Molecular Devices, USA).

**Nascent RNA capture**

Click-iT Nascent RNA Capture Kit (Invitrogen, Carlsbad, CA, USA) was used to biotin-label and capture newly synthesized RNA. In brief, newly synthesized RNA was biotinylated by labeling with 5-ethymyl uridine (EU), and labeled RNA was separated using magnetic streptavidin beads and used for the succeeding qRT-PCR.

**RNA stability Measurement**

De *vivo* synthesis of RNA was blocked by adding actinomycin D (ActD; NobleRyder, China) in cell culture medium. Actinomycin D was used to inhibit cells transcription, and cells were harvested at different time. RNA expression levels were measured by qRT-PCR. The half-time of RNA was determined at the time when its expression level reached to 50% compared to zero time.

**Chromatin immunoprecipitation assay**

The Chromatin immunoprecipitation (ChIP) assay was performed on U251 and U373 cells using Simple ChIP Enzymatic Chromatin IP Kit (Cell signaling Technology, Danvers, Massachusetts, USA) according to the manufacturer’s protocol. Glioma cells were cross-linked with formaldehyde for 10min and terminated with glycine. Cells were harvested in lysis buffer and micrococcal nuclease was used to digest the chromatin. 2% lysates were used as an input reference control and stored at -20℃, and other lysates were incubated with anti-ZBTB4 antibody or normal IgG antibody with rotation. DNA crosslinks were reversed by NaCl and proteinase K and the ChIP DNA was finally purified.

**Tumor xenograft in nude mouse**

For in *vivo* study, Four-week-old athymic nude mice (BALB/c) were purchased from the Beijing HFK Bioscience co.,Ltd. (Beijing, China). All the animal experiments were performed following the Animal Welfare Act and approved by the Ethics Committee of China Medical University. The nude mice were divided into five groups: Control group, MSI2(-) group, SNORD12B(-) group, ZBTB4(+) group and MSI2(-)+SNORD12B(-) +ZBTB4(+) group. For subcutaneous implantation, the stable transfected and expression cells were selected, and 3×10^5^ cells were injected subcutaneously under right axilla area. The volumes of tumor were measured every 5day according to the formula: mm^3^=length×width^2^/2. At 45 days after subcutaneously injection, the mice were sacrificed and the tumors were separated. As for survival study, 3×10^5^ cells were injected into the right striatum. The number of survival mice was recorded every day and survival analysis was performed according to Kaplan-Meier survival curve.

**Gene expression and survival prognosis analysis**

We used the “Pathology Atlas” module of the Human Protein Atlas (https://www.proteinatlas.org/) to obtain the gene expression across all TCGA tumors. The “TCGA analysis” module of UALCAN portal (http://ualcan.path.uab.edu/analysis.html) were utilized to gene expression between glioma and corresponding normal tissues. The “survival analysis” module of GEPIA (http://gepia.cancer-pku.cn/index.html) was used to obtain the overall survival plot.
