# Supplementary material for: Glioma glycolipid metabolism: MSI2–SNORD12B–FIP1L1–ZBTB4 feedback loop as a potential treatment target
Source: Clin Transl Med. 2021 May 12;11(5):e411. doi: 10.1002/ctm2.411 (PMC8114150; doi:10.1002/ctm2.411)
Supplement: Supplementary file 10 — Table S1 The primers of MSI2 mRNA, ZBTB4 mRNA, HK2 mRNA, ACLY mRNA, POLR2E mRNA, KNG1 mRNA, and BRD9 mRNA in quantitative real‐time PCR (qRT‐PCR) Table S2 Sequences of sgRNA for knockdown of SNORD12B TableS3 The short hairpin RNAs against MSI2 and ZBTB4 sites Table S4 Primers used for ChIP experiments [file CTM2-11-e411-s009.docx]

**Tables**

Table S1. The primers of MSI2 mRNA, ZBTB4 mRNA, HK2 mRNA, ACLY mRNA, POLR2E mRNA, KNG1 mRNA, BRD9 mRNA in quantitative real-time PCR (qRT-PCR)

| Primers | Forward | Reverse |
| --- | --- | --- |
| MSI2 | CCCACCATGAGTTAGATTCCAAGACG | GTGTTCGCAGATAACCCGCCTAC |
| ZBTB4-CDS region | GCGTCCTGGCTGCTTCAAGTC | AGGAAGAGGAGGAGGAGGAAGAGG |
| ZBTB4-long UTR region | AATGAGATTGTGGCGACGTGGAG | AAAGAGGGCACGAACTGACAAGAC |
| HK2 | CGACAGCATCATTGTTAAGGAG | GCAGGAAAGACACATCACATTT |
| ACLY | CAGAATCGGTTCAAGTATGCTC | AAGTTTTCCACGACGTTTGATC |
| POLR2E | AAGTACATCCTGGAGCAGTTTC | GTTCTCTCGGAGCTTATATCGG |
| KNG1 | CCTACTCAATTGTGCAAACGAA | GTGGTTGTACAAAATCCTTCCC |
| BRD9 | AAGAAGAAGAAGTCCGAGAAGG | GCATTTTAAGTTCCACCGAGAA |
| β-actin | CCTGGCACCCAGCACAAT | GGGCCGGACTCGTCATAC |

Table S2. Sequences of sgRNA for knockdown of SNORD12B

| sgRNA1 | AAGTCATCATATATGCCAGC |
| --- | --- |
| sgRNA2 | CTGGCATATCAGACAGAAAC |

TableS3 The short hairpin RNAs against MSI2 and ZBTB4 sites

| gene | Site | Sequence(5’-3’) |
| --- | --- | --- |
| MSI2 | #1 | GAATGAAGATGTTGTGGAGAA |
|  | #2 | AGGCACAGAGGGTTTGGCTTT |
|  | #3 | CCCGGATTTGCTCCAAGCTAT |
| ZBTB4 | #1 | GTCTTTGGCTACGCAGTGAAT |
|  | #2 | GCCCAAGCTCAATACACTCAA |
|  | #3 | CCAACTCTTCCTCCACCAATT |

Table S4. Primers used for ChIP experiments

| Gene | Binding site or Control | Forward | Annealing temperature(℃) | Reverse | Annealing temperature(℃) | Product size(bp) |
| --- | --- | --- | --- | --- | --- | --- |
| ACLY | PCR1 | F:TGCCAGACACTGCTGCTTAA | 59.9 | R:TTGTGGCCTGATCGCTTGAG | 60.7 | 204 |
|  | PCR2 | F:GGTTTTGAACTCCTGGACGC | 59.4 | R:GCTCAGGCCTGTAATCCCAT | 59.5 | 207 |
| HK2 | PCR1 | F:AGACAAGGGCAGGGAAGAGA | 60.2 | R:CAAGCAGTCCTCCCACTTCA | 59.6 | 215 |
|  | PCR2 | F:GAAGCTGAGATGGGCGATCA | 59.9 | R:CCTCCTGGGTTCAAGCGATT | 60.0 | 170 |
| MSI2 | PCR1 | F:ATGTGATCCAGGGAAGTCGG | 59.2 | R:GTGGGGCGGAGTTCTAGAAC | 60.1 | 210 |
|  | PCR2 | F:CCTAGGATGGCCTGTGGTTC | 59.8 | R:GTCACTCCAAGCTCCTTCCC | 60.0 | 164 |
